# Multi-omics analyses reveal the biocontrol potential of endophytic *Paenibacillus peoriae* 3-B4 against maize seedling blight

**DOI:** 10.3389/fmicb.2025.1686411

**Published:** 2025-12-12

**Authors:** Yue Hu, Yifan Chen, Shengqian Chao, Yin Zhang, Lili Song, Hui Wang, Yingxiong Hu, Beibei Lv

**Affiliations:** 1 Key Laboratory of Agricultural Genetics and Breeding, Biotechnology Research Institute, Shanghai Academy of Agricultural Sciences, Shanghai, China; 2 Key Laboratory for Safety Assessment (Environment) of Agricultural Genetically Modified Organisms, Ministry of Agriculture and Rural Affairs, Shanghai, China; 3 Shanghai Professional Technology Service Platform of Agricultural Biosafety Evaluation and Testing, Shanghai Academy of Agricultural Sciences, Shanghai, China; 4 Crop Breeding and Cultivation Research Institute, Shanghai Academy of Agricultural Sciences, Shanghai, China; 5 Shanghai Co-Elite Agricultural Sci-Tech (Group) Co., Ltd., Shanghai, China

**Keywords:** *Paenibacillus peoriae*, *Fusarium verticillioides*, *Zea mays*, multi-omics analysis, biocontrol

## Abstract

**Introduction:**

Maize seedling blight, caused by the phytopathogenic fungus *Fusarium verticillioides*, is a common and rapidly spreading disease that reduces grain quality and yield. Control of this pathogen is challenging due to its complex infection process and the development of resistance to chemical pesticides. Biological control agents are therefore recognized as a sustainable and environmentally friendly strategy for the maize seedling blight.

**Results:**

In this study, we isolated and identified *Paenibacillus peoriae* 3-B4 from maize leaves, which exhibited a 59.92% inhibitory rate against *F. verticillioides* in greenhouse co-inoculation conditions. Genome sequencing revealed a 5.91 Mb chromosome containing biosynthetic gene clusters involved in antifungal secondary metabolites, along with genes associated with induced systemic resistance (lSR) and pattern-triggered immunity (PTl). Transcriptomic analysis was performed on maize seedling leaves from a greenhouse experiment, identifying 8,997 differentially expressed maize genes, which were primarily enriched in MAPK signaling and plant–pathogen interactions. 16S rRNA analysis further showed significant shifts in microbial communities, particularly an increase in the abundance of beneficial genera such as *Paenibacillus, Delftia*, and *Corynebacterium*. The combined analysis of key differentially expressed genes (DEGs) and bacterial community profiles suggested coordinated shifts in host transcriptional responses and microbial abundances following *P peoriae* 3-B4 treatment.

**Discussion:**

These findings not only emphasize the importance of endophytic bacteria in plant defense, but also provide new insights into the development of sustainable biological control strategies for maize fungal diseases.

## 1 Introduction

As a highly photosynthetically active C4 crop, maize (*Zea mays L.*) is renowned for its high grain and biomass yields (Majumdar et al., 2021), being extensive utilized and playing an integral role in agriculture. This cereal crop is of both great nutritional and industrial importance globally, functioning as a primary source of food for humans and animals alike, and supplying raw materials for diverse commercial applications (Xing et al., 2023). Accordingly, major maize disease outbreaks have had a detrimental impact on the economy and the food supply in recent years, posing significant health risks to people and livestock (Hernández-Rodríguez et al., 2008). Maize is prone to several fungal diseases, including seedling blight, stalk rot, brown spot, blight, and blackhead disease, all of which severely affect its quality and yield (Pal et al., 2021; Yang et al., 2020). Maize seedling blight, with *F. verticillioides* as its causal agent, is among the most common fungal diseases (Baldwin et al., 2014). This disease typically occurs during the seedling stage and spreads rapidly, extensively damaging maize plants and resulting in significant yield losses; the disease occurrence could rise to as high as 50% in certain fields (Jiang et al., 2022; Meng et al., 2017). The main symptoms include withering of leaf edges and gradual yellowing and drying of leaves from the bottom up (Yang et al., 2017; Dong et al., 2020). From a physiological point of view, seedling blight impairs photosynthesis, leading to stunting, slow growth, poor development, and leaf wilting or yellowing, adversely affecting the overall growth, weakening maize stalks, and making them more susceptible to lodging (Jiang et al., 2022; Zhou et al., 2018).

*Fusarium verticillioides* (Sacc.) Nirenberg (sinônimo, *Fusarium moniliforme*, Sheldon) is the most widespread fungal pathogen causing seedling blight and rot in various tissues of maize at all growth stages (Baldwin et al., 2014; Kommedahl and Windels, 1981). In addition to diminishing crop yield and quality, this pathogen could produce fumonisins, which accumulate in maize and pose a risk to human and animal health (Bacon and Hinton, 2011; Gai et al., 2018). Although traditional chemical control approaches can manage these diseases to some extent, the extensive application of pesticides poses potential risks to the environment, disturbs the ecological equilibrium, and can lead to pathogen resistance (de Fátima Dias Diniz et al., 2021; Nishimoto, 2019). Comparatively, biological control offers distinct advantages. It is safe for human health, exerts lower environmental impacts than synthetic chemical fungicides, and can achieve sustained, long-term control of pathogens (Andrić et al., 2020; Arnaouteli et al., 2021). Therefore, the development of biocontrol strategy is a treading topic in modern plant disease management (He et al., 2021).

To date, biological management strategies to reduce and control fumonisin production in *F. verticillioides* include applying microorganisms (such as probiotics, non-toxic fungal strains, and plant growth-promoting rhizobia), antioxidants, and plant extracts, and utilizing genetically engineered disease-resistant crops (Deepa et al., 2021; Diniz et al., 2022). Harnessing microorganisms to manage crop diseases has been increasingly acknowledged as a viable and sustainable alternative to traditional approaches (van Lenteren et al., 2017). By integrating with plants via, for example, mixing them with roots, seeds, or adding in the soil, biological control agents can effectively target and combat pathogens. Compared to rhizosphere microorganisms, endophytes exhibit superior colonization within plants, and possess inherent colonization advantages (Russo et al., 2016; Tripathi et al., 2022; Wilson, 1995). Moreover, endophytic microorganisms reside within plant tissues without causing any noticeable harm or adverse effects to the host plant (Santos et al., 2022; Zhang et al., 2019). Certain endophytes can have benefits for plants, such as promoting growth and nitrogen fixation and suppressing disease development (Bertalan et al., 2009). Microbial fertilizers and biopesticides developed from plant endophytes and their metabolites hold great potential in sustainable agriculture, and have already contributed notably to advancing sustainable maize production (Firdous et al., 2019). Several bacterial genera have been identified as effective biocontrol agents against various plant diseases (Ayaz et al., 2023). For example, the *Paenibacillus* genus, which contains many endophytic bacterial species, has been shown to stimulate plant growth and inhibit pathogen proliferation (Grady et al., 2016; Yadav et al., 2021). These effects are achieved by inducing plant’s resistance mechanisms or generating inactivating substances (Grady et al., 2016). In light of these findings, the use of *Paenibacillus* strains and isolated antimicrobial compounds to control plant pathogens may reduce our reliance on chemical fungicides (Daud et al., 2019).

This study aims to clarify the mechanisms by which the maize endophyte *Paenibacillus peoriae* 3-B4 contributes to the control of *Fusarium verticillioides*, a major pathogen causing seedling and stalk diseases in maize. Given the limitations and environmental risks associated with chemical fungicides, effective biological control strategies are urgently needed. Although *Paenibacillus* species are known for their growth-promotion and antimicrobial capacities, their specific actions against *F. verticillioides* remain insufficiently understood. Here, we combined greenhouse biocontrol assays, whole-genome sequencing, transcriptome analysis, and microbial diversity studies to investigate how strain 3-B4 may inhibit the pathogen, including its potential to produce antimicrobial compounds, enhance plant defense responses, and influence microbial communities. This work provides mechanistic insights into the biocontrol potential of *P. peoriae* 3-B4 and supports its development as a sustainable alternative for managing maize fungal diseases.

## 2 Materials and methods

### 2.1 Experimental materials

The maize plants used in this study were collected from the Zhuanghang Experimental Station of the Shanghai Academy of Agricultural Science in China (121°23′58″N, 30°53′37″E).

A culture of pathogenic strain *F. verticillioides* 2H12-6 was obtained from the China Specialty Maize Research Centre (Shanghai).

### 2.2 Isolation of endophytic strains and identification of the strain *Paenibacillus peoriae* 3-B4

Maize tissues were surface-sterilized by sequential washing in sterile water, immersion in 75% ethanol, and treatment with 3% sodium hypochlorite, followed by three rinses with sterile water. The sterilized tissues were then cut into small fragments and homogenized in a sterile mortar to obtain a microbial suspension. This suspension was serially diluted (10^−1^, 10^−2^, and 10^−3^), and 200 μL of each dilution was spread onto Nutrient Broth Agar (NB), Potato Dextrose Agar (PDA), and Yeast Extract Peptone Dextrose Agar (YPD), with three replicates per medium. Plates were incubated and examined daily for bacterial and fungal colony development. Emerging colonies were immediately transferred to fresh media for purification.

Taxonomic identification was performed based on 16S rRNA gene sequencing. The 16S rRNA and gyrB gene fragments of *Paenibacillus peoriae* 3-B4 were amplified using genomic DNA as the template (Liu et al., 2021). Primer sequences are listed in Supplementary Table S8. PCR products were sequenced by Sangon Biotech (Shanghai, China). The obtained sequences were aligned and compared using the NCBI BLAST tool. *Paenibacillus* strains were identified through BLAST results, together with *Scopulibacillus daqui* as the outgroup, were included for phylogenetic analysis. A phylogenetic tree was constructed based on 16S rRNA sequences using MEGA 6.0.

Gram staining was additionally conducted to determine whether *P. peoriae* 3-B4 is Gram-positive or Gram-negative. Heat-fixed smears were stained with crystal violet (1 min), treated with iodine (1 min), decolorized with 95% ethanol (20 s), counterstained with safranin (1 min), and examined microscopically.

### 2.3 Whole-genome sequencing and analysis of *Paenibacillus peoriae* 3-B4

Strain *P. peoriae* 3-B4 was cultured in LB broth at 37 °C with shaking, and genomic DNA was extracted using the OMEGA Bacterial DNA Kit following the manufacturer’s instructions. DNA concentration and purity were assessed using a TBS-380 fluorometer, and only high-quality DNA (OD260/280 = 1.8–2.0; > 6 μg) was used for library preparation.

Approximately 3 μg of genomic DNA was used to construct an Illumina paired-end sequencing library. After fragmentation, target inserts were purified, enriched, and PCR-amplified, followed by quality assessment. Sequencing was performed on the Illumina NovaSeq 6000 platform (150 bp × 2; Shanghai BIOZERON Co., Ltd). The whole-genome sequence has been deposited in the NCBI SRA under accession number SRR29484736.

Gene prediction was performed using GeneMark, and functional annotation was conducted through BLASTP searches against the NCBI NR, KEGG, and COG databases.

### 2.4 Screening of antagonistic endophytes against *Fusarium verticillioides* and scanning electron microscopy analysis

The antagonistic activity of the isolated endophytic strains against *F. verticillioides* was assessed using a dual-culture confrontation assay on PDA. A 5-mm mycelial plug taken from the margin of a 7-day-old fungal colony was placed at the center of a 90-mm PDA plate. Bacterial strains were cultured in Luria–Bertani (LB) broth at 37 °C and 200 rpm for 48 h, and the resulting cultures were adjusted to 1 × 10^8^ CFU/mL. A 5-μL aliquot of the bacterial suspension was spotted onto the PDA plate at a distance of 25 mm from the fungal plug. Plates containing only the fungal plug served as controls. All plates were incubated at 28 °C for 7 days (Ali et al., 2021). Antifungal activity was quantified by comparing the radial growth of fungal colonies in treated plates with that of the control. The percentage inhibition of radial growth (PIRG) was calculated as PIRG = ((R₁ − R₂)/R₁) × 100, where R₁ is the fungal colony radius in the control and R₂ is the radius in the direction of the bacterial colony (Raut et al., 2021). Each treatment was performed with three biological replicates.

To examine the interaction between the selected endophytic bacterium and *Fusarium verticillioides* 2H12-6, mycelial samples were collected from the margin of the inhibition zone after 7 days of dual culture. Mycelia grown on PDA alone served as the control. All samples were prepared for scanning electron microscopy (SEM) following a modified version of previously described protocols. Mycelial fragments (≈ 5 × 5 mm) were excised and fixed in 2.5% (v/v) glutaraldehyde in 0.1 M phosphate buffer (pH 7.2) at 4 °C overnight. After three buffer rinses, samples were post-fixed in 1% osmium tetroxide for 1 h and washed again. The tissues were dehydrated through a graded ethanol series (30–100%) and dried using critical point drying. Dried samples were mounted on aluminum stubs and sputter-coated with a 60:40 gold–palladium layer before SEM examination. The coated samples were examined using a TM4000PLUS scanning electron microscope (Hitachi High-Technologies, Japan), and micrographs were captured at appropriate accelerating voltages and magnifications to document morphological alterations in fungal hyphae under antagonistic interaction.

### 2.5 Greenhouse biocontrol experiments

Greenhouse pot experiments were conducted to evaluate the efficacy of *P. peoriae* 3-B4 in controlling maize seedling blight. Maize seeds were surface-sterilized and sown in pots (15 cm diameter) filled with a sterilized soil:sand mixture (2:1, v/v). Seedlings were grown under controlled greenhouse conditions at 25 ± 2 °C with a 16 h light/8 h dark photoperiod and 60–70% relative humidity. Plants were watered regularly to maintain adequate soil moisture and fertilized weekly with Hoagland’s nutrient solution. At the Vegetative stage (V3), seedlings were used for pathogen inoculation.

*F. verticillioides* was cultured on PDA for 14–21 days until full colony development. Conidia were harvested by adding 10 mL of sterile distilled water containing 0.0125% Tween® 80 to the colony surface and gently scraping with a sterile razor blade. The resulting suspension was filtered through sterile cotton gauze to remove mycelial debris, and the conidial concentration was adjusted to 1 × 10^7^ conidia/mL using a hemocytometer. The pathogenic strain *F. verticillioides* 2H12-6 was inoculated onto maize leaves using the pin-prick method (Patil et al., 2017).

Four treatment groups were established. In the CK group, seedlings were wounded in the same manner and sprayed with LB medium. In the P group, seedlings were wounded with a sterilized needle and inoculated with strain 2H12-6. In the B group, seedlings were only sprayed with the *P. peoriae* 3-B4 suspension. In the PB group, seedlings were first sprayed with *P. peoriae* 3-B4 suspension and inoculated with strain 2H12-6 after 12 h. Prior to application, the *P. peoriae* 3-B4 suspension was diluted fivefold to 5 × 10^8^ CFU/mL and uniformly sprayed onto leaf surfaces. Each treatment included 10 replicates, with each replicate consisting of three maize seedlings. LB medium was used as the control.

Disease incidence was recorded 7 days after inoculation, and the disease index was calculated according to the 0–5 rating scale described by Zhao et al. (2022). The disease index and control efficacy were calculated using the following formulas:


$$\text{Disease index}=100\times \frac{\sum (\begin{array}{l} \text{number of infected maize leaves}\\ \times \text{relative disease grade} \end{array})}{\begin{array}{l} \text{total number of maize leaves}\\ \times \text{highest disease grade} \end{array}}$$

$$\text{Control effect}\;(\%)=100\times \frac{\left(\begin{array}{l} \text{disease index of control group}\\ -\text{disease index of treatment group} \end{array}\right)}{\text{disease index of control group}}$$

### 2.6 Transcriptome sequencing and data analysis

Gene expression profiling was performed on maize leaf samples collected from the four treatment groups described above. Five biological replicates per treatment were used (20 samples total). Total RNA was extracted using TRIzol Reagent (Invitrogen), and RNA quantity and integrity were assessed with a NanoDrop spectrophotometer and an Agilent Bioanalyzer 2100.

Three micrograms of RNA per sample were used for library construction. Libraries were purified using the AMPure XP system and quantified with an Agilent high-sensitivity DNA assay before sequencing on the Illumina NovaSeq 6000 platform.

Gene expression levels were normalized as FPKM. Read counts were generated using HTSeq (v0.9.1), and differentially expressed genes (DEGs) were identified with DESeq (v1.30.0) using |log2FoldChange| > 1 and *p* < 0.05 as significance thresholds.

GO enrichment analysis was carried out using topGO with a hypergeometric test (*p* < 0.05), and KEGG pathway enrichment was performed using ClusterProfiler (v3.4.4). Raw RNA-seq data have been deposited in the NCBI SRA under accession numbers SRR29873971–SRR29873980.

### 2.7 Quantitative real time-PCR analysis

To validate the RNA-seq results, 11 DEGs associated with plant disease resistance were selected for quantitative real time-PCR (qRT-PCR) analysis. Gene-specific primers are listed in Supplementary Table S9. Total RNA was extracted using TRIzol, and the same RNA samples used for RNA-seq were used for qRT-PCR.

cDNA was synthesized using the PrimeScript™ 1st Strand cDNA Synthesis Kit and used as template for qPCR. Relative gene expression was calculated using the 2^–ΔΔCt^ method with β-actin as the internal reference. Each sample was analyzed with at least three technical replicates.

### 2.8 16S rRNA sequencing and bioinformatic analysis

DNA was extracted from maize leaves in the CK group (*n* = 5) and the B group (*n* = 5) under greenhouse conditions using the OMEGA Soil DNA Kit (M5635-02, Omega Bio-Tek, Norcross, GA, United States). The V3–V4 region of the bacterial 16S rRNA gene was amplified by PCR. PCR products were purified with VAHTS DNA Clean Beads (Vazyme, Nanjing, China) and quantified using the Quant-iT PicoGreen dsDNA Assay Kit (Invitrogen, Carlsbad, CA, United States). Equimolar amplicons were pooled and sequenced (2 × 250 bp) on the Illumina NovaSeq 6000 platform at Shanghai Personal Biotechnology Co., Ltd.

The microbiome bioinformatics analysis was performed using QIIME2 2019.4 (Bolyen et al., 2019). QIIME2 and the R package (v3.2.0) were mostly used to analyze sequence data. Sequences were then quality filtered, denoised, merged and chimera removed using the DADA2 plugin. Non-singleton amplicon sequence variants (ASVs) were aligned with mafft and used to construct a phylogeny with fasttree2. Alpha diversity indices at the ASV level, including the Chao1 richness estimator, Shannon diversity index, and Simpson index, were computed based on the ASV table in QIIME2 and presented as box plots. All the raw reads have been deposited under the BioProject accession number PRJNA1134884 in the NCBI BioProject database.

## 3 Results

### 3.1 Isolation, screening, and classification of antagonistic endophytic strains against *Fusarium verticillioides*

**Figure 1:**
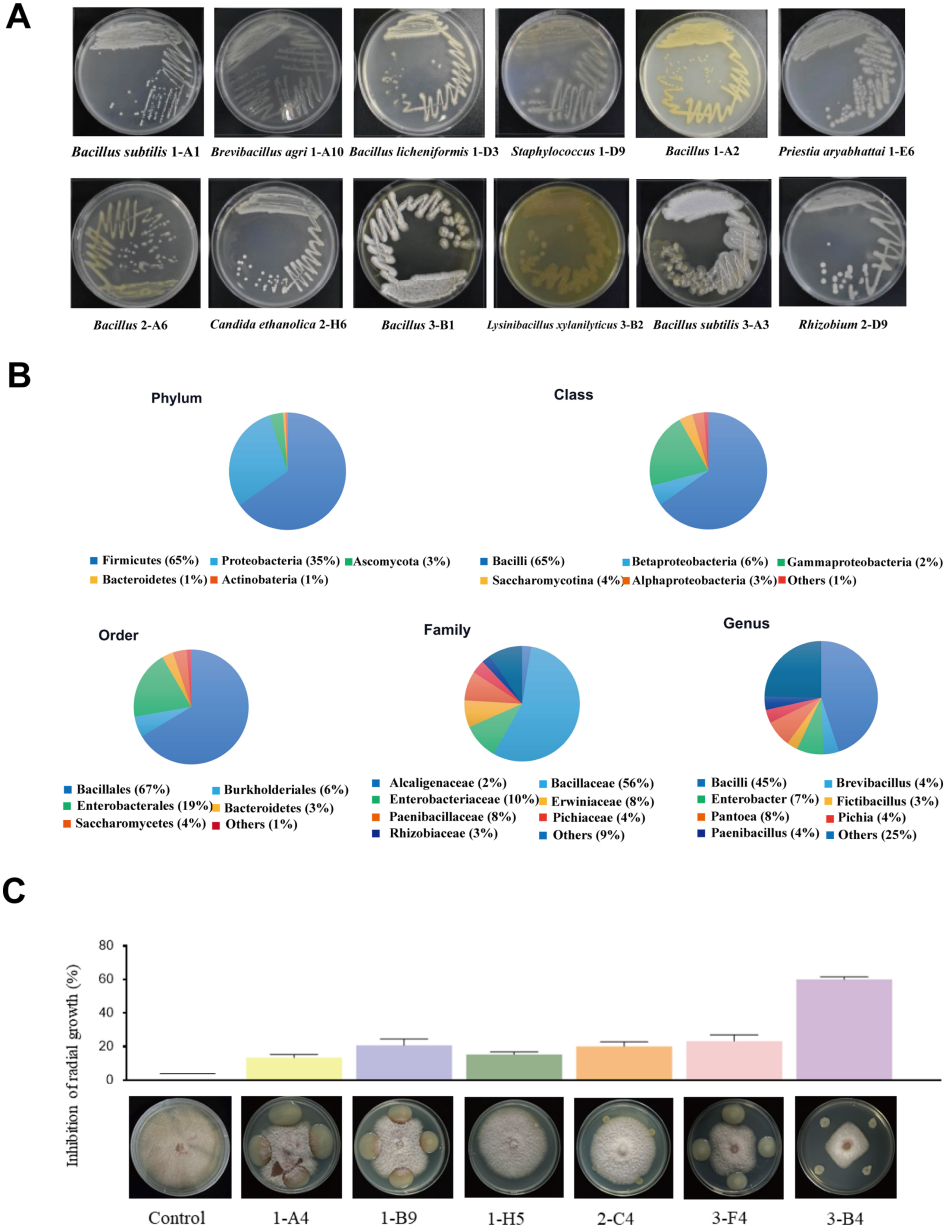
Isolation, identification, and antagonistic screening of endophytic bacteria from maize. **(A)** Colony morphology of isolated bacteria. **(B)** Distribution and diversity analysis of 148 maize endophytic bacterial strains at different taxonomic levels. **(C)** Fungal inhibition assays of certain strains against *Fusarium verticillioides* 2H12-6.

A total of 147 bacterial strains and one actinomycete strain were isolated from *Zea mays L.* in this study. Genomic DNA was isolated from these strains, followed by amplification of the 16S rRNA fragment. Species classification was completed after sequencing (Supplementary Table S1). The bacteria were numbered, preserved, and recorded for further analysis. Colony morphological characteristics are shown in Figure 1A and Supplementary Table S2. Statistical analysis of these strains revealed that *Bacillus*, *Pantoea*, *Enterobacter*, *Paenibacillus*, and *Brevibacillus* accounted for 45, 8, 7, 4, and 4% of the total isolates, respectively. The isolated strains belong to 26 genera from 16 families. Among these, high abundance genera were *Bacillus*, *Enterobacter*, and *Pantoea* (Figure 1B), suggesting that these three genera were dominant in the culturable endophytic flora of maize tissues.

The antifungal effects of the isolated endophytic bacteria were verified through plate confrontation assays. Strain 3-B4 exhibited the greatest effective inhibition on *F. verticillioides* 2H12-6 growth, as indicated by the smallest growth area of fungal colonies (32.9 mm in diameter), and the largest inhibition rate (59.92%) (Supplementary Table S3). In contrast, when co-cultured with *F. verticillioides* 2H12-6, the growth areas of *F. verticillioides* in the antagonistic assays with strains 1-A4, 1-B9, and 3-F4 were significantly larger than those of strain 3-B4, and the fungal mycelium showed no obvious inhibition (Figure 1C).

### 3.2 Morphological and molecular identification of *Paenibacillus peoriae* 3-B4

The electron micrograph showed that the colonies of 3-B4 were yellow in color, with intact borders. Upon Gram staining, the colonies exhibited a reddish purple coloration, confirming that strain 3-B4 was a Gram-negative bacterium (Supplementary Figures S1B,C).

The BLAST search and sequence comparison using the 16S rRNA sequence of strain 3-B4 in the GenBank nucleotide database revealed a high sequence similarity to *P. peoriae*, indicating a close phylogenetic relationship with other *Paenibacillus* species. The molecular identification aligned with our morphological and physiochemical characterization, confirming that strain 3-B4 belongs to the species *P. peoriae* (Supplementary Figure S1A).

### 3.3 Inhibition of the morphological structure of *Fusarium verticillioides* by *Paenibacillus peoriae* 3-B4

**Figure 2:**
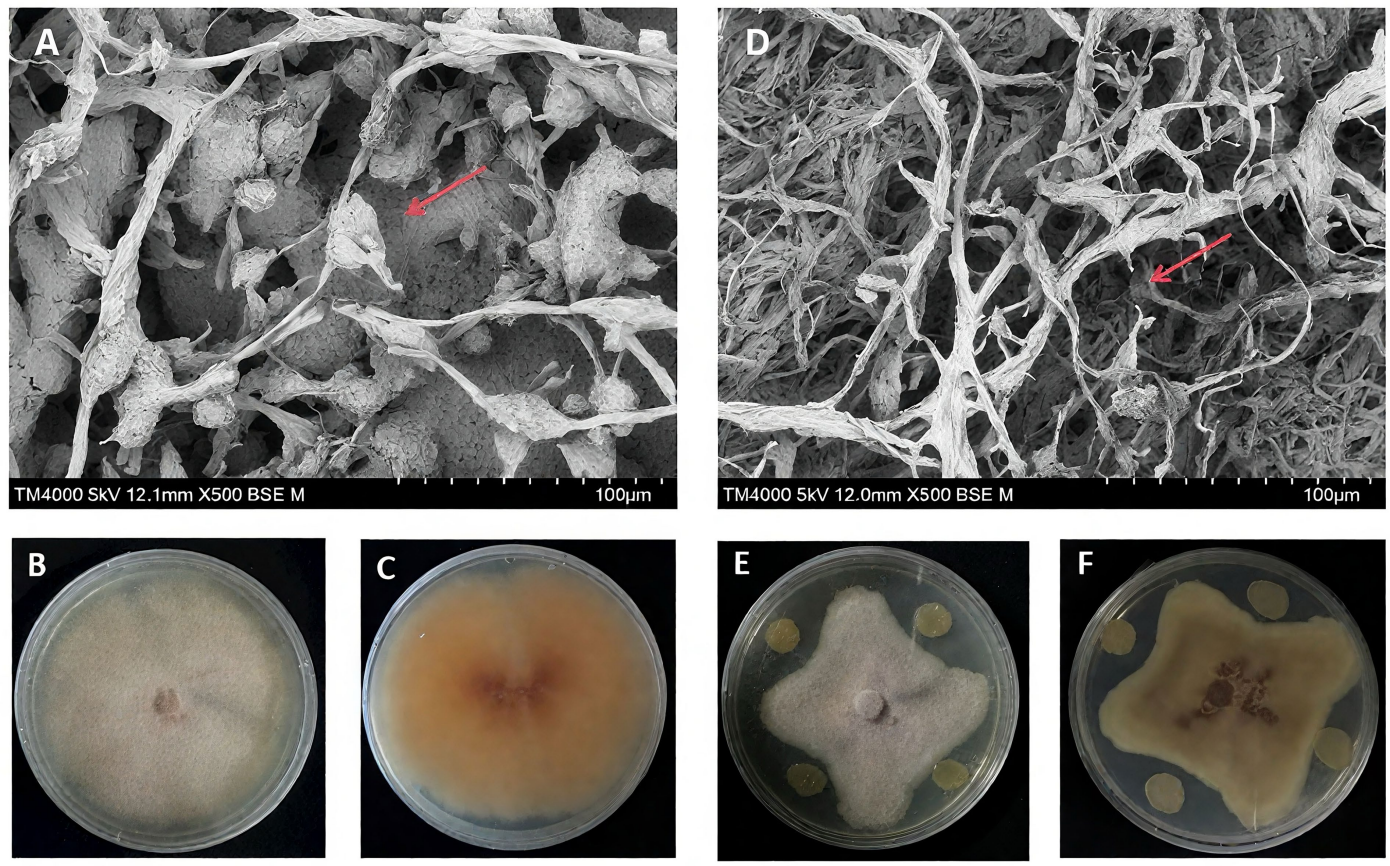
Morphological alterations of *Fusarium verticillioides.* **(A–C)** *Fusarium verticillioides* 2H12-6 in single-strain culture. **(D–F)** *Fusarium verticillioides* 2H12-6 cultured with *Paenibacillus peoriae* 3-B4. **(A)** The arrows indicate the presence of swollen tissue structures. **(D)** The arrows denote hyphal breakage.

The antifungal activity of *P. peoriae* 3-B4, which produces fusaricidin with potent broad-spectrum activity, was studied to assess its impact on the fungal pathogen structure (Zhao et al., 2022). The antifungal activity of *P. peoriae* 3-B4, which produces fusaricidin with potent broad-spectrum activity, was studied to assess its impact on the fungal pathogen structure. Morphological observations using electron microscopy revealed that during the infection process, *F. verticillioides* 2H12-6 exhibited tumor-like swollen structures in pure cultures, representing specific morphological changes occurring (Figures 2A–C). In contrast, during the antagonistic interaction between *F. verticillioides* 2H12-6 and *P. peoriae* 3-B4, the tumor-like structures were not observable, the number of spores decreased, and the hyphae showed breakage (Figures 2D–F). This suggests that *P. peoriae* 3-B4 may inhibit the infection structures of *F. verticillioides* hyphae, thereby further controlling pathogen growth.

### 3.4 Biocontrol efficacy of *Paenibacillus peoriae* 3-B4 against maize seedling blight

**Figure 3:**
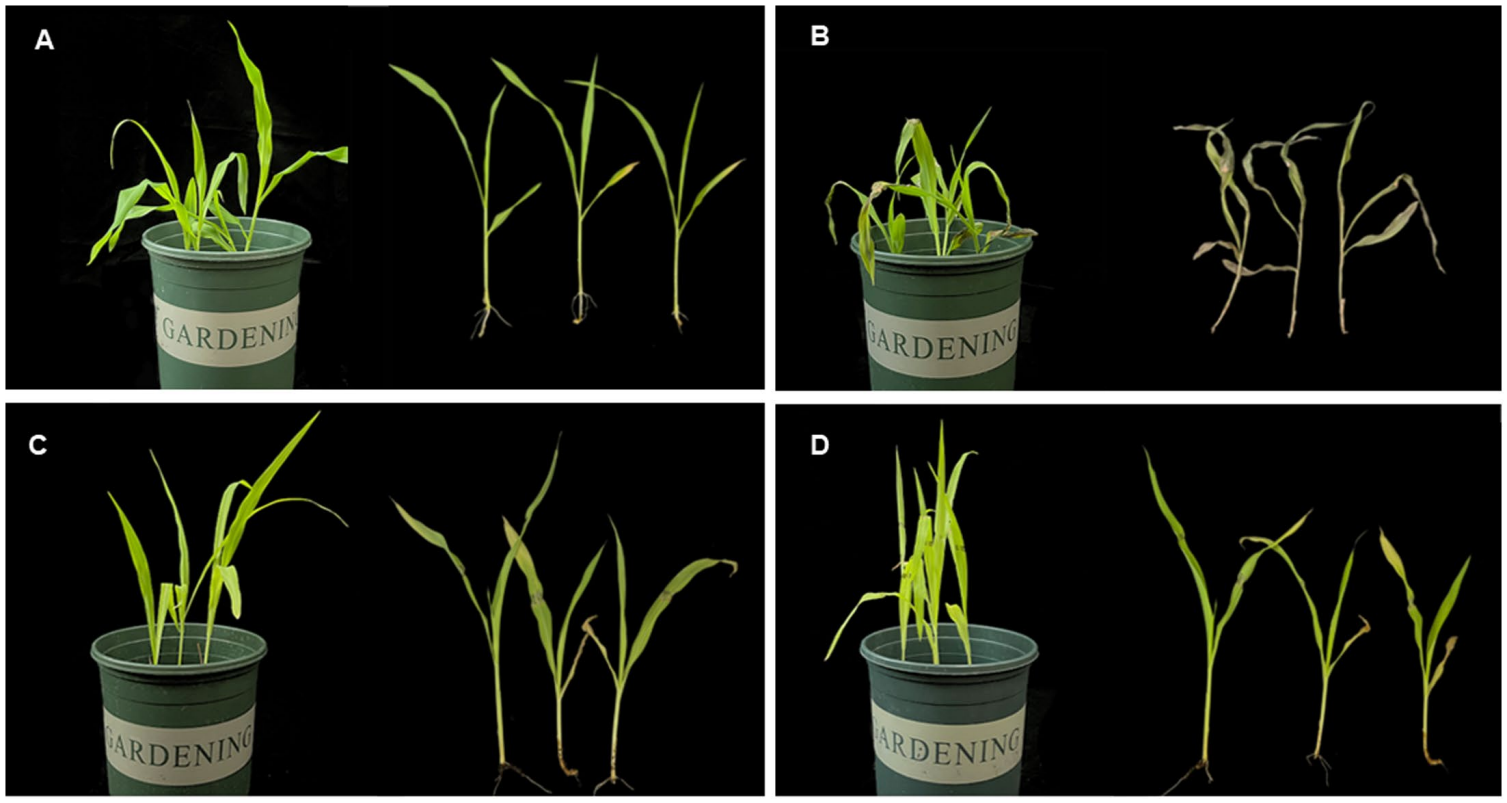
Biocontrol efficacy of *Paenibacillus peoriae* 3-B4 in maize seedlings. **(A)** Maize seedlings sprayed with LB liquid medium. **(B)** Maize seedlings inoculated with pathogenic fungus *F. verticillioides* 2H12-6. **(C)** Maize seedlings treated with biocontrol bacterium *P. peoriae* 3-B4. **(D)** Maize seedlings inoculated with pathogenic fungi after pretreatment with biocontrol bacterium *P. peoriae* 3-B4.

The efficacy of *P. peoriae* 3-B4 as a biocontrol agent against maize seedling blight was validated through pot experiments. On plants inoculated only with fungal pathogen *F. verticillioides* (Group P), leaf spots rapidly expanded, leaves curled and shriveled, and the disease extended to the whole seedling, which dried up and died (Figure 3B). In contrast, when the *P. peoriae* 3-B4 was applied to pretreat the maize leaves (Group PB), reduced disease symptoms were observed, with suppressed spots and no visible leaf yellowing (Figure 3D). The growth of plants sprayed only with the biocontrol bacterium (Group B), as shown in Figure 3C, was comparable to the control group (Group CK), as shown in Figure 3A, indicating that plant growth wasn’t affected by the *P. peoriae* 3-B4’s application. Seven days after inoculation with *F. verticillioides*, the morbidity of maize seedlings in group P exceeded 80%. At this point, the disease condition index was calculated. The pre-treatment group (Group PB) that received *P. peoriae* 3-B4 exhibited a significant decrease in the occurrence and severity of maize leaf disease and in maize seedling blight index compared to Group CK. The P group exhibited a disease index of 80.34%, while the PB group demonstrated a significantly reduced disease index of 20.56%. Compared to the P group, the disease index was reduced by 59.78% at 7 days after inoculation with *F. verticillioides*, resulting in a biocontrol efficacy of 77.23%.

### 3.5 General biological and genomic characteristics of *Paenibacillus peoriae* 3-B4

**Figure 4:**
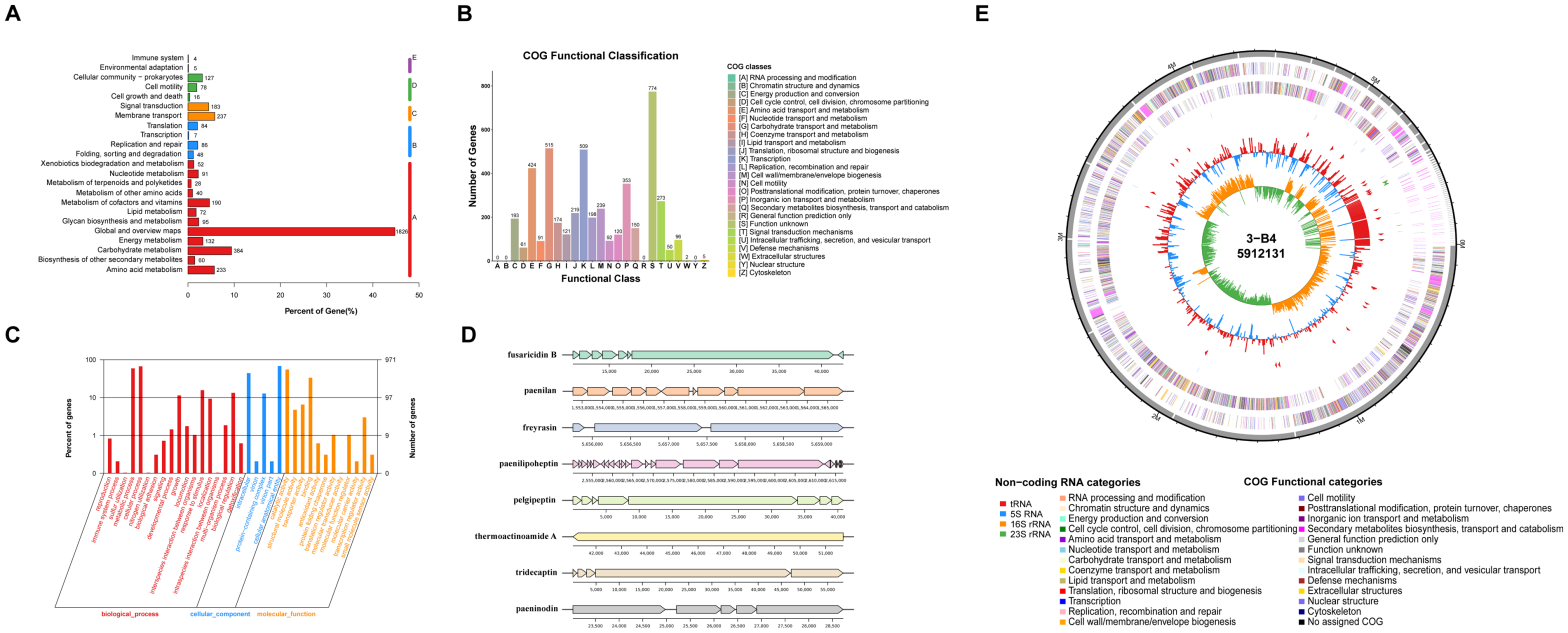
Whole genome analysis of *Paenibacillus peoriae* 3-B4. **(A)** KEGG functional annotation classification statistics. **(B)** COG functional annotation classification statistics. **(C)** GO functional annotation classification statistics. **(D)** Cluster of secondary metabolite-related genes annotated by antiSMASH. Each band represents a gene, and the color and shape indicate the functional category or expression of the gene. Genomic locations indicated by numerical labels in the figure. The direction of gene transcription is indicated by an arrow. **(E)** Graphical circular map of the *P. peoriae* 3-B4 genome. The outermost Layer 1 indicates the genome size. Layer 2 and 3 show coding DNA sequences (CDS) on the positive and negative strands, with different colors representing different COG functional categories. Layer 4 represents rRNA and tRNA. Layer 5 illustrates GC content, where outward red parts indicate regions with higher GC content than the genome average, and inward blue parts indicate regions with lower GC content, with peak height reflecting the deviation from the average.

*P. peoriae* 3-B4’s whole genome comprises a circular DNA structure with 5,839,239 base pairs and a mean G + C content of 45.51%. An overview of the assembly details and genomic characteristics is presented in Figure 4E and Supplementary Tables S4, S5. The genome revealed 5,383 predicted genes, including 17 ribosomal RNA operons and 79 tRNAs (Supplementary Table S6).

A total of 2,731 functional genes were identified and mapped using the Kyoto Encyclopedia of Genes and Genomes (KEGG) database. These genes were categorized into five groups of KEGG metabolic pathways. The largest share of annotated genes, amounting to 1826, was linked to cellular process metabolic pathways. Among these, most were concentrated in Global and overview maps, Carbohydrate metabolism, and Amino acid metabolism (Figure 4A; Supplementary Figure S2).

Gene function annotation of *P. peoriae* 3-B4 using the Gene Ontology (GO) database is shown in Figure 4C. Among the three GO categories, Biological process contained the largest collection of enriched genes, particularly metabolic and cellular processes. This category was followed by Molecular function, where catalytic activity and binding-related genes were the most abundant, while genes related to Molecular transducer activity were the least represented. Figure 4B presents the Clusters of Orthologous Groups of Proteins (COG) annotation analysis for *P. peoriae* 3-B4. Of all coding genes, 4,229 were annotated to the COG database, accounting for approximately 78.56%. These genes were distributed in 24 homologous gene clusters. After removing 774 genes with unknown functions, three major homologous gene clusters with a large number of predicted genes were obtained: carbohydrate transport and metabolic functions (515 genes, 12.18%), transcriptional functions (509 genes, 12.03%), and amino acid transport and metabolic functions (424 genes, 10.03%).

### 3.6 Genes/gene clusters involved in antibiotic synthesis and plant resistance induction

*Paenibacillus* species were shown to have potent broad-spectrum antifungal activity (Yuan et al., 2022). In the genome sequence of *P. peoriae* 3-B4, genes associated with secondary metabolism were identified in the genome sequence of *P. peoriae* 3-B4 using the AntiSMASH database to explore potential secondary metabolites linked to antifungal activity. Gene clusters involved in several pathways of biosynthesis were identified, four of which encode non-ribosomal peptide synthetases (NRPSs). Notably, fusaricidin B, paenilan, pelgipeptin, paeninodin, and tridecaptin exhibited significant antifungal activity against fungal pathogens (Figure 4D) (Yuan et al., 2022; Zavaliev and Dong, 2024).

The *P. peoriae* 3-B4 genome was analyzed using KEGG to identify key genes involved in induced systemic resistance (ISR). These included 10 ISR-related genes and 3 genes linked to pattern-triggered immunity (PTI), all of which were analyzed for their roles in resistance inducer biosynthesis. The characteristics of these genes in *P. peoriae* IBSD35, GXUN15128, and HS311 were compared using the NCBI database (Table 1). The results indicated that the genomes of these four *P. peoriae* strains contained key genes related to volatile organic compounds in ISR, including those encoding the production of 2,3-butanediol, methanethiol, isoprene, and peptidoglycan, with their sequences in the *P. peoriae* 3-B4 genome showing over 95% homology to the corresponding sequences in strains IBSD35, GXUN15128, and HS311.

**Table 1:** Comparative analysis of genes related to the synthesis of resistance inducer strains.

Gene name	Plant resistance type	Resistance inducers	Location	Identity (%)		
				*P. peoriae IBSD*35	*P. peoriae* GXUN15128	*P. peoriae* HS311
alsD	ISR	2,3-Butanediol	1,291,944–1,291,198	99.6	99.6	97.58
bdhA	ISR	2,3-Butanediol	425,759–426,820	99.43	99.71	99.71
alsS	ISR	2,3-Butanediol	1,293,804–1,292,107	96.09	96.28	96.28
alsR	ISR	2,3-Butanediol	912,647–911,748	99.67	97.99	97.66
ilvN	ISR	2,3-Butanediol	541,932–542,417	100	100	100
ispE	ISR	Isoprene	18,527–19,390	99.3	99.65	98.94
ispF	ISR	Isoprene	50,295–49,819	96.2	95.57	96.2
metE	ISR	Methanethiol	310,364–312,682	98.45	98.58	98.58
metH	ISR	Methanethiol	327,895–324,515	99.83	99.74	99.91
mmuM	ISR	Methanethiol	535,045–534,098	98.41	99.37	98.73
tuf	PTI	Elongation factor Tu	27,186–25,996	98.99	100	100
dacA	PTI	Peptidoglycan	34,599–35,555	100	100	100
dacA	PTI	Peptidoglycan	63,246–61,858	99.78	98.48	98.48

### 3.7 Transcriptomic profiles of *Zea may L.* upon *Paenibacillus peoriae* 3-B4 application

We constructed a total of 10 libraries for CK and B, with raw reads of 3.70–4.97 Gb and approximately 3.60–4.83 Gb of clean reads. The values for Q20 and Q30 were above 96.19 and 93.35% (Supplementary Table S7). Comparison efficiency is defined as the percentage of mapped reads to clean reads. In our mapping process, the comparison efficiency was greater than 70% and these mapped reads were fully assembled, showing a high-matching rate to the reference genome and no signs of contamination. The ratio efficiency is over 88.92%, and more than 96.50% of reads were located in exonic regions. The unigenes annotated from the NR database were aligned with various species, with 95.02% of them identified as belong to maize, which is generally consistent with previous reports. Overall, these results demonstrate that the sequencing was of high quality and met the necessary criteria for further analysis.

**Figure 5:**
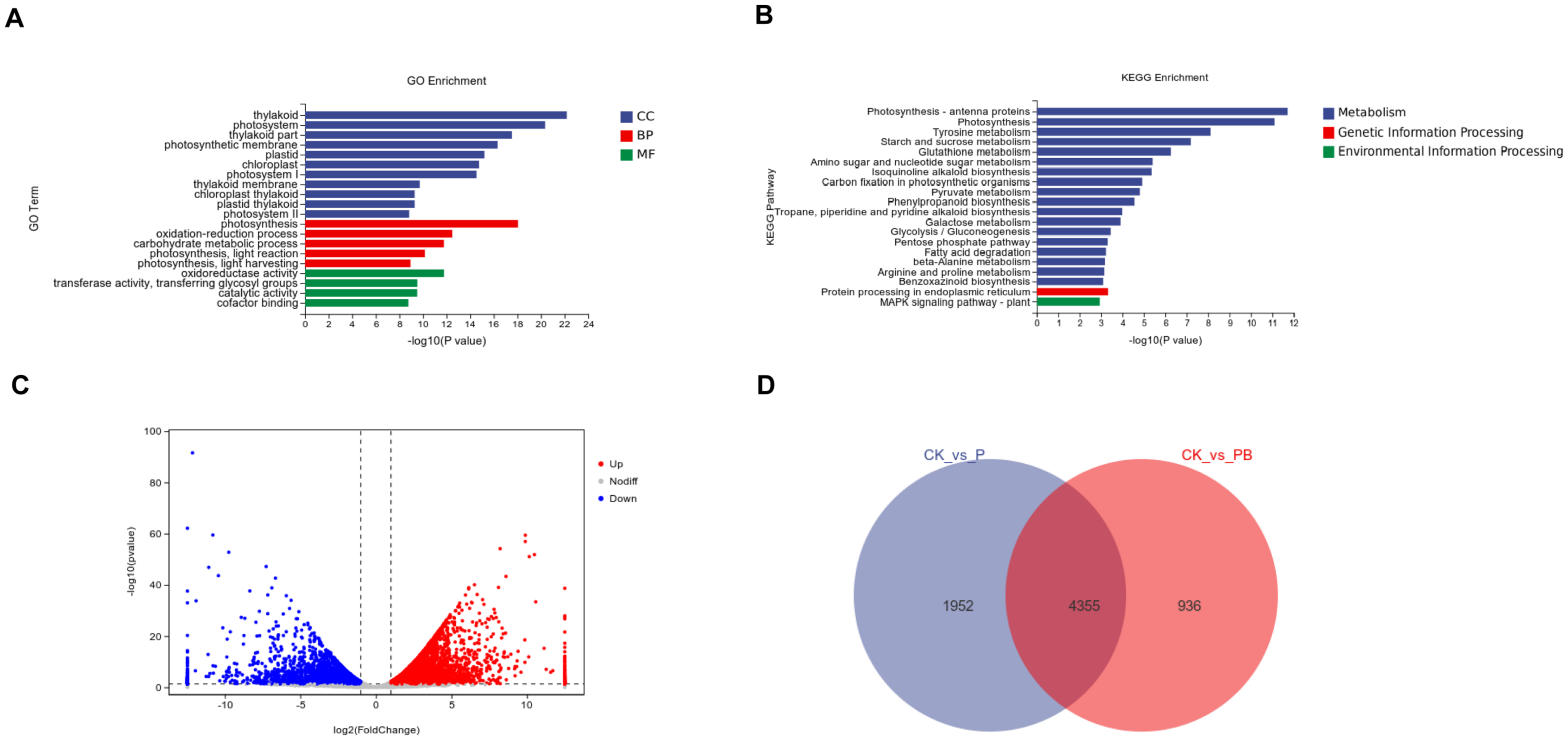
Transcriptional level analysis of *Paenibacillus peoriae* 3-B4 on maize leaves. **(A)** GO enrichment plot of differentially expressed genes. **(B)** KEGG enrichment plot of differentially expressed genes. **(C)** Volcano map of differentially expressed genes. **(D)** Venn diagram of unique differentially expressed genes in CK vs. P and CK vs. PB comparisons. **(A)** The abbreviations represent the gene ontology categories: CC (cellular component, blue bars), BP (biological process, red bars), and MF (molecular function, green bars).

In our differential expression analysis, 8,997 differentially expressed genes (DEGs) were detected when comparing CK with B. Among these, 5,059 showed upregulated, while 3,938 showed downregulated (Figure 5A), indicating significant changes in maize gene expression after *P. peoriae* 3-B4 application. The volcano plot in Figure 5C illustrates the statistical significance and fold-change of gene expression differences.

Eleven genes were randomly chosen from the identified DEGs and analyzed by quantitative PCR (qRT-PCR) to confirm the RNA sequencing (RNA-Seq) results. β-actin served as a control gene. The qPCR results showed a similar expression pattern to the transcriptome data’s expression count values for each gene, thereby validating the RNA-Seq-generated expression results (Supplementary Figure S3).

#### 3.7.1 GO functional enrichment analysis of the DEGs

In the GO enrichment analysis of DEGs between the CK and B groups, there are 4 molecular functions terms, 5 biological process terms, and 11 cellular component terms (Figure 5A). Within Cellular Components (CC), the most significantly enriched terms were thylakoid membrane system-related categories, including “thylakoid” (GO:0009579), “photosystem” (GO:0009521), and “thylakoid part” (GO:0009534), collectively representing 38% of cellular component DEGs. These were followed by “photosynthetic membrane” (GO:0034357) and “plastid” (GO:0009536), which we clarify both refer to specialized membrane structures within chloroplasts. Notably, the term “plastid” in this context specifically refers to chloroplast subcompartments rather than other plastid types. Biological Process (BP) DEGs were predominantly associated with photosynthetic pathways, including “photosynthesis, light reaction” (GO:0019684) and “photosynthesis, light harvesting” (GO:0009765). In terms of Molecular Functions (MF), dominant categories included “oxidoreductase activity” (GO:0016491) and “transferase activity” (GO:0016740), particularly those transferring glycosyl groups. This analysis indicates that *P. peoriae* 3-B4 primarily influences maize leaf metabolism by modulating photosynthetic machinery, redox homeostasis and carbohydrate metabolism pathways.

#### 3.7.2 Biological pathways mediating the effects of *Paenibacillus peoriae* 3-B4

To better understand the the biological pathways influenced by *P. peoriae* 3-B4, KEGG functional annotation analysis was conducted on all DEGs identified by RNA-Seq. The results revealed that 8,997 DEGs showed enrichment in 112 KEGG pathways (Figure 5B). In these pathways, DEGs were most significantly enriched in protein processing in the endoplasmic reticulum and plant–pathogen interactions (Table 2). According to the KEGG annotation results, we selected the following five KEGG pathways with significantly enriched DEGs for subsequent analysis: photosynthesis-antenna protein (zma00196), mitogen-activated protein kinase (MAPK) signaling pathway—plant (zma04016), ABC transporters (zma02010), protein processing in endoplasmic reticulum (zma04075), and plant–pathogen interaction (zma04626).

**Table 2:** KEGG pathway enrichment of DEGs.

Pathway ID	Pathway	DEGs	*P*-value	Adjust *P*-value
zma04075	Protein processing in endoplasmic reticulum	104	0.003728596	0.017123921
zma04626	Plant-pathogen interaction	85	0.025746243	0.086284707
zma00520	Amino sugar and nucleotide sugar metabolism	79	1.17212E−05	0.000215533
zma00500	Starch and sucrose metabolism	76	6.21356E−06	0.000154096
zma00940	Phenylpropanoid biosynthesis	74	0.003291745	0.015699092
zma04016	MAPK signaling pathway - plant	67	0.004918376	0.021030297
zma00010	Glycolysis / Gluconeogenesis	66	0.001364274	0.010565242
zma00195	Photosynthesis	59	6.00827E−16	7.45025E−14
zma00480	Glutathione metabolism	57	2.15614E−06	6.68402E−05
zma00564	Glycerophospholipid metabolism	55	0.001973083	0.012017907
zma00620	Pyruvate metabolism	54	1.21672E−05	0.000215533
zma00710	Carbon fixation in photosynthetic organisms	49	3.22756E−05	0.000444686
zma00230	Purine metabolism	47	0.019766614	0.072090005
zma00620	Pyrimidine metabolism	41	0.000431791	0.004662085
zma00710	Glyoxylate and dicarboxylate metabolism	39	0.010652104	0.044028696

**Figure 6:**
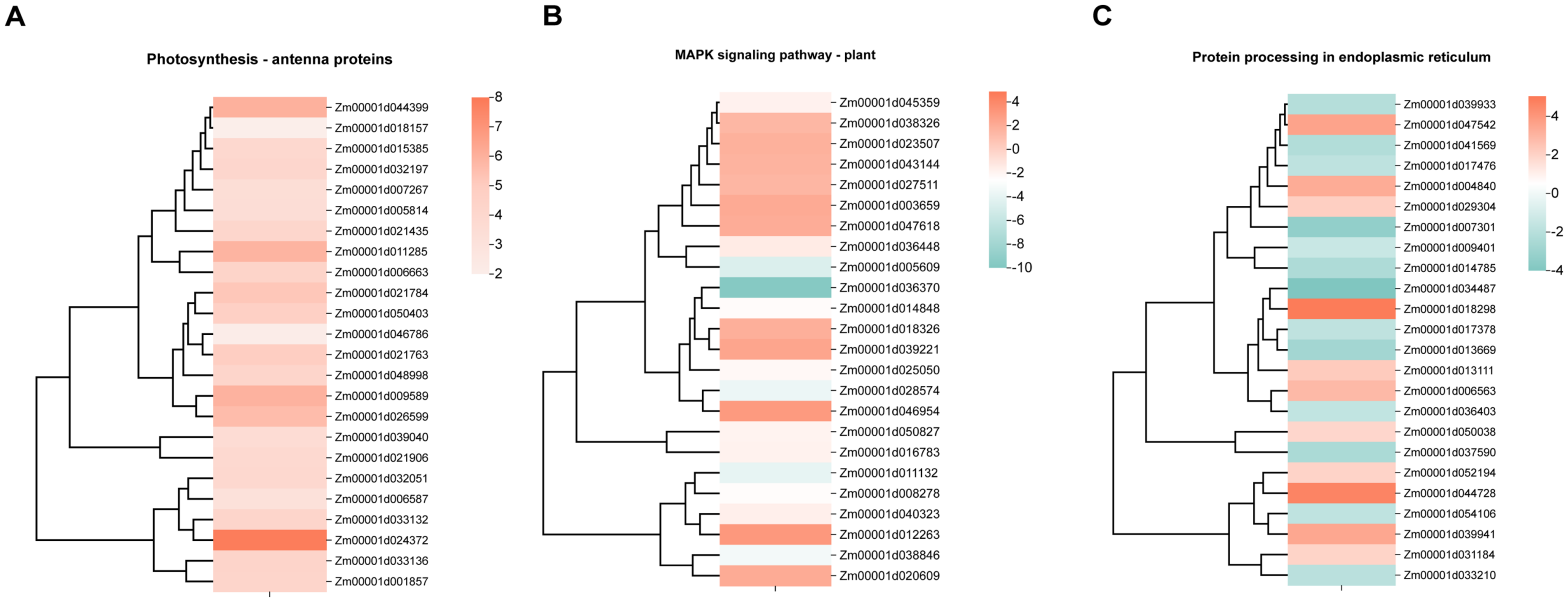
Heatmap showing expression profiles of differentially expressed genes involved in plant defense–related pathways. The red color represents up-regulation, and the blue represents down-regulation. **(A)** Genes involved in photosynthesis-antenna protein. **(B)** Genes involved in MAPK signaling pathway—plant. **(C)** Genes involved in protein processing in endoplasmic reticulum.

In the photosynthesis-antenna protein pathway, the expression levels of 27 DEGs exhibited a notable increase in the biocontrol-treated leaves (Group B) relative to the control leaves (Group CK) (Figure 6A). Among these DEGs, 5 Lhca genes, 5 Lhcb genes, and 7 Cab genes were upregulated, while 1 LHBC gene was downregulated. The capture and transfer of light energy is the main function of these genes. The Lhca gene family encodes Light-harvesting Complex I (LHCI) proteins, a series of pigment-binding membrane proteins in Plant Photosystem I (PSI). In total, 67 DEGs were identified as being associated with the MAPK signaling pathway, with 25 showing increased expression and 42 showing decreased expression (Figure 6B). Notably, the MPK3, SAPK4, Rbohe, and CAT2 genes exhibited increased expression, while the MPK1, MPK5, MPK12, WRKY24, and PYL4 genes exhibited decreased expression. Among the MKK5 genes, two had increased expression, and one had decreased expression, indicating tha their different functions in MAPK signaling pathway. In the plant-pathogen interaction pathway, 85 DEGs were identified, with 40 upregulated and 45 downregulated. Differential expression was identified for calcium-binding protein (CML)-encoding genes, PTI1-like tyrosine-protein kinase (PTI) genes, 3-ketoacyl-CoA synthase (KCS), and Calcium-dependent protein kinase (CPK) genes. Furthermore, decreased expression was observed for five Plant Immune Receptor (RPM1) genes and five WRKY transcription factors. The functionality of these genes is of vital importance in plant responses to various environmental stresses. RPM1 is involved in recognizing pathogen effector-induced modifications and triggering immune responses. WRKY transcription factors, on the other hand, act as regulators of gene expression, modulating the transcription of genes involved in stress responses and defense mechanisms. In protein processing in the endoplasmic reticulum pathway, 104 DEGs were identified, with 41 exhibiting increased expression and 63 showing decreased expression (Figure 6C). Among these, eight DnaJ/40 heat shock proteins demonst rated increased expression. Additionally, five HSP70 genes exhibited increased expression, and they contribute to protein folding and the stress response; whereas four PDI genes showed decreased expression, and these genes are associated with protein folding and the maintenance of redox homeostasis.

#### 3.7.3 Potential functional genes and transcription factors related to pathogen resistance

In order to identify potential disease-related genes involved in maize responses to *F. verticillioides* infection and *P. peoriae* 3-B4 treatment, we compared DEGs between CK vs. B and CK vs. PB (Figure 5D). The CK vs. B comparison revealed 1,952 DEGs, while CK vs. PB revealed 936 unique DEGs. A total of 4,355 DEGs were shared between the two comparisons, suggesting that these genes may participate in responses common to both treatments, including enrichment in plant–pathogen interaction, MAPK signaling, and plant hormone signal transduction pathways.

The expression of an RPP13-like gene, encoding a nucleotide-binding site leucine-rich repeat (NBS-LRR) resistance protein, was not altered by *F. verticillioides* infection alone but increased significantly following *P. peoriae* 3-B4 application, suggesting a possible activation of plant defense responses. The Zm00001d042111 gene, encoding the salicylic acid (SA) signaling regulator Nonexpressor of Pathogenesis-Related Genes 1 (NPR1), showed a 3.69-fold and 1.65-fold increase in B and P groups, respectively, compared to CK. There was no significant change observed in the PB group, which may reflect potential modulation of NPR1-mediated SA signaling during co-inoculation. It is worth noting that NPR1 represents a pivotal node in the interconnection between SA signaling and PR gene expression and is of significant importance for plants to acquire systemic acquired resistance (Cao et al., 1994; Roetschi et al., 2001).

Overall, these transcriptional changes highlight candidate defense-related genes that could contribute to the observed biocontrol effects, but further functional validation will be required to confirm their roles.

### 3.8 Shifts in the microbial community composition of maize leaves treated with the biocontrol bacterium *Paenibacillus peoriae* 3-B4

Through 16S rRNA gene sequencing of leaf samples, we analyzed the impact of the biocontrol bacterium *P. peoriae* 3-B4 on the microbial communities of maize leaves. By comparing bacterial species and relative abundances in the CK and B groups, our aim was to identify the key microbial taxa that may interact with or be influenced by the *P. peoriae* 3-B4. We obtained 133,801 and 137,725 effective sequence reads from the CK and B groups, respectively, by 16S rRNA diversity sequencing. A total of 1,372 amplicon sequence variants (ASVs) were identified, with 66 ASVs shared between the two groups.

**Figure 7:**
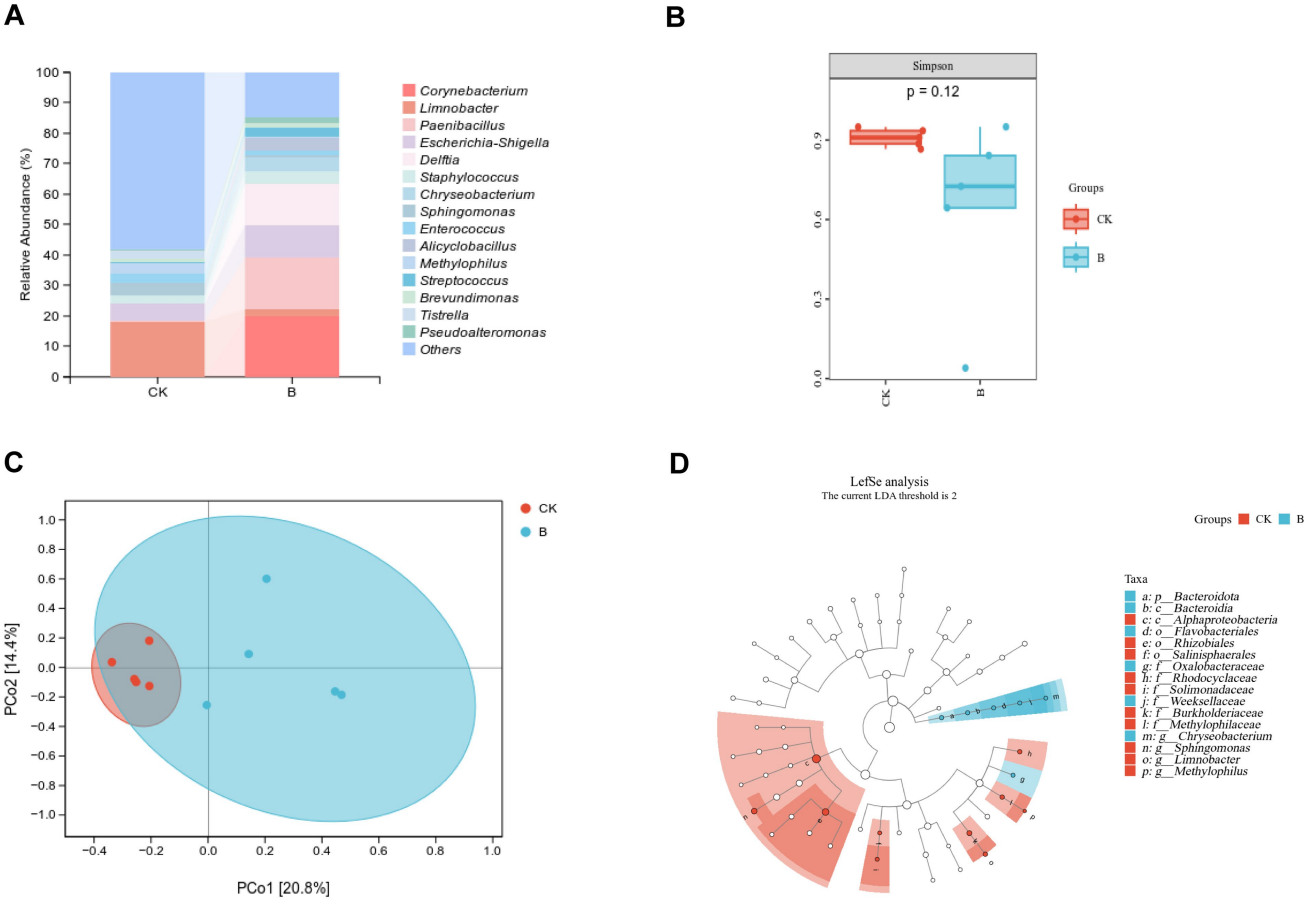
Comparison of microbial community composition between CK and B groups: **(A)** Relative abundance of bacteria community, **(B)** Simpson’s diversity index, **(C)** PCoA score plot, and **(D)** LEfSe analysis.

Microbial composition analysis revealed that the 5 most predominant phyla in the control group were Proteobacteria (67.60%), Firmicutes (28.01%), Actinobacteriota (2.08%), Bdellovibrionota (2.01%), and Acidobacteriota (0.19%). In contrast, the biocontrol treatment group (B group) displayed a different pattern, with the top 5 phyla being Proteobacteria (41.23%), Firmicutes (32.87%), Actinobacteriota (20.51%), Bacteroidota (4.71%), and Myxococcota (0.54%). *Limnobacter* (17.92%), *Escherichia-Shigella* (5.46%), *Sphingomonas* (4.14%), *Enterococcus* (2.87%), and *Staphylococcus* (2.68%) were the top five genera in the control group, while *Corynebacterium* (19.96%), *Paenibacillus* (17.29%), *Delftia* (13.26%), *Escherichia-Shigella* (10.60%), and *Chryseobacterium* (4.71%) dominated the biocontrol treatment group (Figure 7A). We used the Simpson’s index for α-diversity. In general, a higher value of the traditional Simpson’s index indicates lower diversity, therefore a higher Simpson’s index in the CK group suggests lower actual diversity compared to the B group (Figure 7B). This implies that the B group harbored a more diverse bacterial community following *P. peoriae* 3-B4 treatment. For β-diversity, the differences and similarities (Bray-Curtis index) of the maize microbial community between the control group (CK) and the groups treated with the biocontrol bacterium (B) were analyzed using Principal Coordinate Analysis (PCoA). As shown in Figure 7C, the samples from the CK and B groups showed distinct clusters.

In the CK and B groups, dominant microbial biomarkers were found at the phylum to genus level using linear discriminant analysis effect size (LEfSe). LEfSe difference analysis demonstrated that the abundance of bacterial taxa, such as *Bacteroidota*, *Bacteroidia*, *Flavobacteriales*, *Oxalobacteraceae*, *Weeksellaceae*, and *Chryseobacterium*, was more abundant in group B than in CK (Figure 7D). These changes imply possible involvement in modulating the microbiome during *P. peoriae* 3-B4 colonization, even though the specific mechanisms of these taxa require further functional validation. In conclusion, *P. peoriae* 3-B4 application resulted in an increase of the maize bacterial community diversity, which could contribute to alterations of the community’s function after *P. peoriae* 3-B4 treatment.

### 3.9 Interactions between microbes and DEGs under the biocontrol treatment

To investigate the relationship between microbes and host genes in maize treated with the biocontrol bacterium *P. peoriae* 3-B4, we conducted genus-level associations between the CK and B groups by comparing bacterial relative abundances to the expression of the top 200 DEGs.

**Figure 8:**
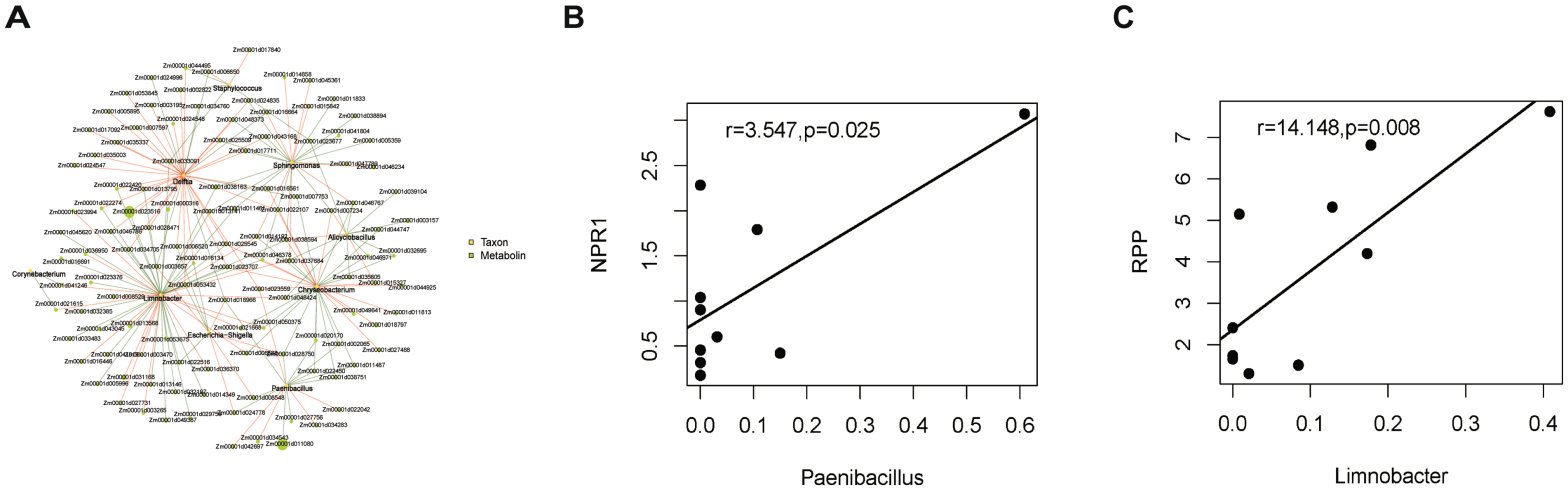
Interactions between the microbes and DEGs related to plant disease resistance. **(A)** Network plot of significant microbe–gene correlations (top 200 DEGs in transcriptome). Red lines indicate a positive correlation and green lines indicate a negative correlation. **(B)** Correlation plots of NPR1 and *Paenibacillus* combinations. **(C)** Correlation plots of RPP and *Limnobacter* combinations.

As illustrated in Figure 8A, a significant positive correlation (*p* < 0.05) was observed between the majority of DEGs and the relative abundance of *Delftia*, while *Limnobacter* exhibited an enrichment of negative associations (*q* < 0.05). The results of the analysis indicated positive associations between CCT and *Chryseobacterium* and *Alicyclobacillus* (*q* < 0.05), and bZIP correlated positively with *Delftia* and *Chryseobacterium* (*q* < 0.05). Conversely, HSP genes were found to be negatively associated with *Limnobacter* and *Sphingomonas* (*q* < 0.05).

In addition to the global gene-microbe correlation analyses, we highlighted the relationships between several representative genes and microbial taxa, both of which have previously been linked to host disease resistance (Figures 8B,C). NPR1, a key activator of systemic acquired resistance, showed a strong positive correlation with *Paenibacillus* (*r* = 3.547, *p* = 0.025). The NPR1 gene is required to activate and regulate the plant’s defense mechanisms against multiple pathogens (Zavaliev and Dong, 2024). Furthermore, RPP, an Arabidopsis disease resistance gene (Rose et al., 2004), showed a positive correlation with *Limnobacter* (*r* = 14.148, *p* = 0.008). The proteins encoded by RPP genes are typically members of the nucleotide-binding site-leucine-rich repeat (NBS-LRR) protein family, the function of which is integral to the plant immune system (Bittner-Eddy et al., 1999; Jones et al., 2024). These associations show that the bacterial community shift caused by *P. peoriae* 3-B4 is statistically linked to defense-related transcriptional reprogramming in maize leaves.

## 4 Discussion

Biocontrol using in planta bacteria represents a natural and sustainable method for controlling various plant diseases. Endophytic bacteria, capable of colonizing specific plant cells or tissues and exhibiting resilience to environmental stress, hold significant potential as alternatives to traditional biocontrol agents (Medison et al., 2022; Watts et al., 2023). Microbial fertilizers and biopesticides derived from endophytes and their metabolites are increasingly regarded as promising disease control solutions for future agriculture (Hilário and Gonçalves, 2022; Watts et al., 2023).

Previous morphological observations using electron microscopy have revealed that the formation of nodular structures on *F. verticillioides* hyphae during its infection process is primarily associated with infection structures resembling appressorium-like formations at the hyphal tips (Ha et al., 2023). Different from *P. peoriae* RP51 (Ali et al., 2021), scanning electron microscopy imaging in our study showed that the mycelial morphology changed, but that the mycelium of *F. graminearum* was shrunk and distorted when confronted with *P. peoriae* RP51. In research on the antimicrobial properties of *P. peoriae* IBSD35, findings revealed that the pathogen colonies gradually disappeared after the application of the biocontrol bacterium *P. peoriae* IBSD35 (Ngashangva et al., 2022). These findings indicate that *Paenibacillus* species inhibit pathogens through a range of distinct inhibitory mechanisms.

The *Paenibacillus* genus is well-known for generating antimicrobial compounds such as fusaricidins, pelgipeptin, surfactins, and polymyxins (Ngashangva et al., 2022; Grady et al., 2016). Based on the antiSMASH database, *P. peoriae* 3-B4 produces eight antimicrobial metabolites. Paenilan, a conserved ribosomally synthesized peptide found in *Paenibacillus* species worldwide, acts as a novel class I antibiotic with antimicrobial effects against Gram-positive bacteria, including *Bacillus*, *Micrococcus*, and *Paenibacillus* species (Park et al., 2017). Tridecaptins, another non-ribosomal antimicrobial peptide synthesized by *Bacillus* and *Paenibacillus* species (Kato et al., 1978; Kato et al., 1979), exhibits effective mechanistic activity against Gram-negative bacteria with a low risk of resistance development, potentiating its safe application for environment (Ballantine et al., 2019; Cochrane et al., 2014). In addition to their antimicrobial properties, fusaricidin B and paeninodin inhibit the growth of phytopathogenic fungi (Li et al., 2024; Zhu et al., 2016). Fusaricidin produced by NRPSs has great industrial application potential, given previous findings of its specific inhibitory actions against Gram-positive bacteria and phytopathogenic fungi (Lal and Tabacchioni, 2009). The antifungal mechanism of fusaricidin involves permeation and disruption of the cell membrane (Jiang et al., 2022). Although paeninodin is a novel lasso peptide classified under ribosomally synthesized and post-translationally modified peptides (RiPPs), it demonstrates broad antimicrobial and antiviral activity (Lal and Tabacchioni, 2009).

In the genome of *P. peoriae* 3-B4, we identified 13 genes associated with ISR and PTI, and they exhibited high levels of homology (>95% similarity) with other *P. peoriae* strains, suggesting that *P. peoriae* 3-B4 may function in a similar manner to other species within the *Paenibacillus* genus in inducing resistance. Biological control agents can produce systemic resistance inducers, including volatile organic compounds such as methanethiol, isoprene, 2,3-butanediol, and ethyl butyrate, which are released into the environment to counteract pathogen invasion and thus provide protection (Grady et al., 2016).

After *P. peoriae* 3-B4 application, significant changes in the transcriptome of maize occurred, specifically the overexpression of NPR1, bZIP, MYB, LRR, and WRKY. These changes may induce a set of defense mechanisms to combat pathogens directly and indirectly. GST involved in antioxidant defense mechanisms, as it reduces the reactive oxygen species (ROS) levels through its peroxidase activity (Mittler et al., 2022) and thereby protects cells from oxidative damage and enhancing disease resistance (Zhang et al., 2024). Therefore, we hypothesize that the biocontrol effects of *P. peoriae* 3-B4 on maize are conferred through inducing specific GST genes and other above mentioned functionally relevant genes.

Moreover, DEGs in the control group and the group treated with *P. peoriae* 3-B4 were mainly enriched in five signaling pathways: plant hormone signaling, MAPK signaling pathway, plant–pathogen interactions, protein processing in the endoplasmic reticulum, and ABC transporter synthesis. These pathways play a crucial role in corn’s defensive response against diseases. The plant hormone signaling and MAPK signal pathways are also induced in tomato plants infected with Trichoderma afroharzianum TM24 (Zhao et al., 2021). The results are in line with the findings of Shao et al. (2023), in which most DEGs in maize involved in plant hormone signal transduction, MAPK signal pathway, and plant-pathogen interaction pathways. The MAPK signaling pathway plays a crucial part in plant resistance to pathogen infection (Meng and Zhang, 2013). This pathway involves a cascade reaction of a number of kinases that rapidly respond to external pathogen attack by transducing signals to the nucleus and activating disease resistance genes (Pitzschke et al., 2009). By regulating multiple defense mechanisms, the MAPK pathway enhances plant defenses against a wide range of pathogens, such as bacteria, fungi, and viruses (Zhang et al., 2007). In addition, the MAPK pathway regulates the transduction of phytohormonal signals (e.g., the ones of jasmonic acid and ethylene), which further orchestrate local and systemic defense responses in plants (Wu et al., 2007). The MAPK pathway’s downstream components, known as WRKY transcription factors, have the ability to regulate the transcription of genes associated with different defensive reactions in plants. In rice, WRKY67 can induce the expression of genes involved in plant defense, and RNA-Seq analysis has shown that its upregulation significantly enhances resistance to bacterial blight (Liu et al., 2018). The genes involving the MAPK pathway were also induced in our study, with their increased expression potentially contributing to enhanced pathogen resistance in maize.

Microbiota is widely recognized for its functions in regulating plant immunity, promoting plant growth, and maintaining overall host health. Current research into the application of biocontrol agents has mainly centered on the alteration of community composition of phyllosphere microbiota, rhizosphere microbiota, and in the soil. However, the in planta microbiota has been relatively little studied. Jia et al. (2023) and Wang et al. (2021) found that the microbial community structure and the relative abundance of some beneficial genera significantly changed in the biocontrol treatment groups compared with CK in the phyllosphere and soil, indicating that BCA can enhance the composition of microbial communities. In this study, Simpson’s diversity index was much higher in the CK group than the *P. peoriae* 3-B4 treatment group. Additionally, the treatment group exhibited higher levels of beneficial microbiota, including *Paenibacillus*, *Delftia*, *Chryseobacterium*, and *Pseudomonas*. Of these, *Paenibacillus* is commonly found in plants and performs nitrogen fixation, promoting plant growth and biocontrol properties. *Delftia* can degrade various pollutants, acts as a biological control, and promotes plant growth through multiple mechanisms, such as producing siderophores and antimicrobial compounds (Yin et al., 2022). *Chryseobacterium* contributes to the health of plant root systems through nutrient cycling, delivering plant growth-regulating hormones, and exhibiting biocontrol capabilities against plant pathogens (Jung et al., 2023). Furthermore, in combination with the diversity analysis results, we concluded that *P. peoriae* 3-B4 treatment increases microbiota diversity, especially beneficial microorganisms, in maize. These results collectively indicate that biocontrol agent treatment not only alters the microbial community composition in the phyllosphere, rhizosphere, and soil but also affects the internal microbiome of the host plant. In turn, this influences the composition and role of microbial populations across different plant niches, thereby playing a role in pathogen resistance.

**Figure 9:**
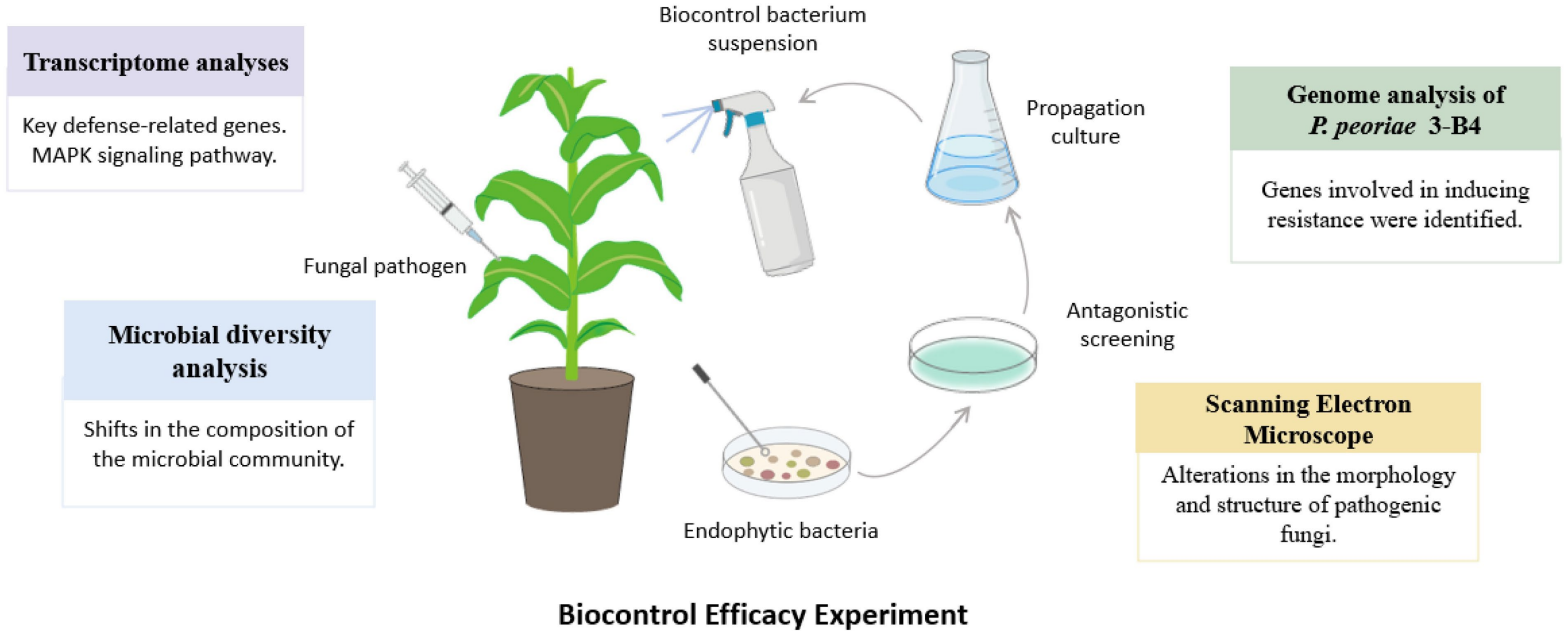
Overview of the multi-omics and experimental approaches used to investigate the biocontrol activity of *Paenibacillus peoriae* 3-B4 against maize seedling blight.

In our study, we used multi-omics analysis to determine the biocontrol effectiveness of *P. peoriae* 3-B4 application on *F. verticillioides* (Figure 9). Genes that induce resistance have been identified in the genome of *P. peoriae* 3-B4. When activated, these genes trigger the plant’s immune response, which includes plant hormone signaling, ROS burst, activation of MAPK and defense genes, shifts in transcriptional regulation, and secondary metabolite synthesis (Piasecka et al., 2015; Tsuda and Katagiri, 2010; Yu et al., 2017; Zhao et al., 2011). Following the application of *P. peoriae* 3-B4, transcriptome analysis revealed a significant upregulation of disease resistance-related pathways and gene expression levels in maize. NPR1, a key regulatory protein required for plant defense, was found to be significantly upregulated through transcriptome data screening. NPR1, a key regulatory protein that is vital for plant defense, was identified as significantly upregulated. Additionally, 16S rRNA analysis revealed that *P. peoriae* 3-B4 treatment not only caused significant changes in microbial community composition, with a higher Simpson’s index than the control group, but also increased beneficial microbial community diversity, including *Paenibacillus*, *Delftia*, and *Corynebacterium*. Further transcriptome and microbial diversity analyses revealed a statistically significant positive relationship between NPR1 expression and beneficial microbes, particularly Paenibacillus species. These correlations point to a potential role for beneficial microbes in improving plant immune responses. Overall, the upregulation of resistance-associated genes, increased NPR1 transcript levels, and enrichment of plant defense-related taxa suggest that *P. peoriae* 3-B4 treatment causes transcriptional changes in disease resistance pathways as well as shifts in microbiome composition. While these associations are correlative and need to be functionally validated, they suggest that *P. peoriae* 3-B4 has the potential to influence host–microbe interactions, which could contribute to improved plant immunity.

## Data Availability

The datasets presented in this study can be found in online repositories. The names of the repository/repositories and accession number(s) can be found in the article/Supplementary material.
