# Supplementary material for: Multi-omics analyses reveal the biocontrol potential of endophytic Paenibacillus peoriae 3-B4 against maize seedling blight
Source: Front Microbiol. 2025 Dec 12;16:1686411. doi: 10.3389/fmicb.2025.1686411 (PMC12741152; doi:10.3389/fmicb.2025.1686411)
Supplement: Supplementary file 1 [file Table_1.docx]

Supplementary Material

**Supplemental Table 1. Statistical table of endophytic bacterial species of maize.**

|  | Name | Strain Number |
| --- | --- | --- |
| 1 | *Candida ethanolica* | 2-H4, 2-H5, 2-H6 |
| 2 | *Acidovorax avenae* | 1-C5, 1-C8 |
| 3 | *Acidovorax oryzae* | 2-C4 |
| 4 | *Acinetobacter sp.* | 1-F13 |
| 5 | *Agrobacterium larrymoore* | 3-B5, 3-C2 |
| 6 | *Alcaligenes aquatilis* | 1-B7 |
| 7 | *Bacillus badius* | 1-D5 |
| 8 | *Bacillus cereus* | 1-A3, 1-A8, 1-B1, 1-B2, 1-E1, 1-F2, 1-H8, 2-A4, 2-D1, 2-D4, 2-D6, 2-D8, 2-E4, 2-E7, 2-E11, 3-C1, 3-D2, 3-D3, 3-D6, 3-E2 |
| 9 | *Bacillus inaquosorum* | 2-E1 |
| 10 | *Bacillus licheniformis* | 1-D3, 1-D6, 1-F3, 1-D8 |
| 11 | *Bacillus pacificus* | 1-D1, 2-D3, 3-D1 |
| 12 | *Bacillus paramycoides* | 1-B5, 2-A2 |
| 13 | *Bacillus pumilus* | 1-H5 |
| 14 | *Bacillus* sp. | 1-B3, 1-D7, 1-D12, 1-F9, 1-F14, 1-H7, 2-A1, 2-A3, 2-D2, 2-E2, 2-E10, 2-A6, 3-B1, 3-F1, 3-H1, 1-A2, 1-E5, 1-A6 |
| 15 | *Bacillus subtilis* | 1-A1, 1-A7, 1-D2, 1-D11, 1-E2, 2-A5, 2-A7, 2-E3, 2-F6, 2-A8, 3-A3, 3-E1 |
| 16 | *Bacillus thuringiensis* | 1-F1, 2-D5, 3-D5 |
| 17 | *Bacillus toyonensis* | 1-H1, 3-A1 |
| 18 | *Brevibacillus agri* | 1-A10, 1-A14 |
| 19 | *Brevibacillus nitrificans* | 2-D14 |
| 20 | *Brevibacillus* sp. | 1-A4, 1-A12, 1-A13, 1-C7 |
| 21 | *Burkholderia gladioli* | 2-E6, 2-E12 |
| 22 | *Chryseobacterium* sp. | 2-D11 |
| 23 | *Enterobacter asburiae* | 1-F12, 2-F2, 3-F5 |
| 24 | *Enterobacter mori* | 1-F5, 1-F7 |
| 25 | *Enterobacter* sp. | 1-F11, 2-F3, 3-F3, 3-F4 |
| 26 | *Enterococcus faecium* | 3-A2 |
| 27 | *Fictibacillus* sp. | 1-A9, 1-A11, 1-D4, 1-D10, 1-F4 |
| 28 | *Klebsiella oxytoca* | 2-F1 |
| 29 | *Kosakonia cowanii* | 2-G3, 2-G4 |
| 30 | *Lysinibacillus pakistanensis* | 1-F6, 1-F8 |
| 31 | *Lysinibacillus xylanilyticus* | 3-B2 |
| 32 | *Margalitia camelliae* | 2-D10 |
| 33 | *Metabacillus indicus* | 1-F10 |
| 34 | *Methylobacterium* sp. | 2-D12 |
| 35 | *Micrococcus yunnanensis* | 1-B9 |
| 36 | *Paenalcaligenes* sp. | 1-C1, 1-C3 |
| 37 | *Paenalcaligenes suwonensis* | 1-B4 |
| 38 | *Paenibacillus peoriae* | 3-B4, 3-C3 |
| 39 | *Paenibacillus polymyxa* | 1-D14, 2-D13, 2-E8 |
| 40 | *Pantoea ananatis* | 2-B1, 2-B2, 2-B4, 2-C1, 2-C2, 1-B10, 3-B3, 3-B6 |
| 41 | *Pantoea* sp. | 1-B6, 2-B3 |
| 42 | *Pichia* sp. | 2-H2 |
| 43 | *Priestia aryabhattai* | 1-E3, 1-E6, 1-H6, 1-H10 |
| 44 | *Priestia megaterium* | 1-H9 |
| 45 | *Providencia* sp. | 1-E4 |
| 46 | *Providencia stuartii* | 1-C2 |
| 47 | *Pseudomonas oryzihabitans* | 1-C6 |
| 48 | *Rhizobium* sp. | 2-D9 |
| 49 | *Rossellomorea marisflavi* | 1-H3, 1-H4 |
| 50 | *Staphylococcus capitis* | 2-E5 |
| 51 | *Staphylococcus* sp. | 1-D9 |

**Supplemental Table 2. Morphology of isolated maize endophytic bacterial colonies.**

|  | Strain number | Colony morphology |
| --- | --- | --- |
| A | 1-A1 | The colony is circular, white, matte, smooth-surfaced, opaque, easy to lift, convex, and with serrated edges. |
| B | 1-A10 | The colony is circular, white, glossy, smooth-surfaced, transparent, easy to lift, flat, and with entire margins. |
| C | 1-D3 | The colony is circular, milky white, non-glossy, with a rough surface, opaque, easily lifted, raised, and has serrated edges. |
| D | 1-D9 | The colony is circular, white, non-glossy, with a smooth surface, opaque, easily lifted, flat, and has wavy edges. |
| E | 1-A2 | The colony is circular, yellow, glossy, with a smooth surface, opaque, easily lifted, raised, and has entire edges. |
| F | 1-E6 | The colony is circular, white, non-glossy, with a smooth surface, opaque, easily lifted, flat, and has entire edges. |
| G | 2-A6 | The colony is oval, pale yellow, glossy, with a smooth surface, semi-transparent, raised, and has entire edges. |
| H | 2-H6 | The colony is circular, milky white, non-glossy, with a smooth surface, opaque, easily lifted, flat, and has entire edges. |
| I | 3-B1 | The colony is circular, white, non-glossy, with a rough surface, opaque, viscous, raised, and has entire edges. |
| J | 3-B2 | The colony is circular, pale yellow, non-glossy, with a rough surface, semi-transparent, flat, and has serrated edges. |
| K | 3-A3 | The colony is circular, white, non-glossy, with a rough surface, opaque, viscous, raised, and has entire edges. |
| L | 2-D9 | The colony is circular, milky white, glossy, with a smooth surface, semi-transparent, raised, and has entire edges. |

**Supplemental Table 3. Inhibition rates of different strains against *Fusarium verticillioides* 2H12-6 by maize endophytic strains**

| Treatment | R1 (mm) | R2 (mm) | Inhibition rate (%) |
| --- | --- | --- | --- |
| CK | 82.0±1.20 | - | - |
| 1-A4 | - | 70.9±1.56 | 13.50±0.02 |
| 1-H5 | - | 69.4±1.25 | 15.33±0.02 |
| 2-C4 | - | 65.4±2.16 | 20.20±0.03 |
| 3-F4 | - | 62.9±3.07 | 23.33±0.04 |
| 1-B9 | - | 65.0±3.04 | 20.77±0.04 |
| 3-H1 | - | 57.7±3.20 | 29.59±0.04 |
| 3-E2 | - | 55.0±0.70 | 32.89±0.01 |
| 3-A3 | - | 49.0±1.72 | 40.20±0.02 |
| 3-E1 | - | 56.4±1.10 | 31.22±0.01 |
| 1-H3 | - | 60.8±1.55 | 25.81±0.02 |
| 3-B1 | - | 61.5±2.44 | 25.00±0.03 |
| 3-D1 | - | 54.7±1.23 | 33.25±0.02 |
| 3-C3 | - | 45.3±2.82 | 44.76±0.03 |
| 3-F2 | - | 62.7±2.14 | 23.54±0.03 |
| 3-H2 | - | 69.9±1.47 | 14.76±0.02 |
| 3-B4 | - | 32.9±1.29 | 59.92±0.02 |

**Supplemental Table 4. Statistics of genome assembly results**

| Sample ID | *Paenibacillus peoriae* 3-B4 |
| --- | --- |
| Total scaffolds | 69 |
| Total base | 5912131 |
| No. of large scaffords (>1kbp) | 49 |
| Bases in large scaffolds | 5898312 |
| Largest length | 1445181 |
| Scaffold N50 | 517276 |
| Scaffold N90 | 90675 |
| G+C content | 45.51% |
| N rate | 0% |

**Supplemental Table 5. Genetic Information Statistics**

| Sample ID | *Paenibacillus peoriae* 3-B4 |
| --- | --- |
| Gene number | 5383 |
| Gene total length | 5180256 |
| Gene average length | 962 |
| Gene density genes per kb | 0.910 |
| GC content in gene region | 46.4% |
| Gene/Geonme | 87.6% |
| plus Gene number | 2810 |
| minus Gene number | 2573 |

**Supplemental Table 6. ncRNA statistics**

| SampleID | ncRNA type | number | average length(bp) | total length(bp) | length/Genome(%) |
| --- | --- | --- | --- | --- | --- |
| *P. peoriae* 3-B4 | tRNA | 79 | 77 | 6091 | 0.1% |
| *P. peoriae* 3-B4 | 5S rRNA | 12 | 115 | 1383 | 0.02% |
| *P. peoriae* 3-B4 | 16S rRNA | 3 | 1290 | 3871 | 0.06% |
| *P. peoriae* 3-B4 | 23S rRNA | 2 | 2820 | 5641 | 0.09% |

**Supplemental Table 7. Overview of RNA-Seq Data**

| Sample | RawData(bp) | CleanData(bp) | Q20(%) | Q30(%) | Uniquely_Mapped (%) |
| --- | --- | --- | --- | --- | --- |
| CK1 | 39031788 | 38010358 | 96.37% | 93.68% | 33095601 (95.96%) |
| CK2 | 41351282 | 40231124 | 96.27% | 93.50% | 34466693 (94.54%) |
| CK3 | 39376482 | 38348946 | 96.33% | 93.60% | 33544878 (95.53%) |
| CK4 | 41760170 | 40669212 | 96.38% | 93.71% | 35168533 (95.02%) |
| CK5 | 39399152 | 38235846 | 96.21% | 93.35% | 33491073 (95.96%) |
| B1 | 40560026 | 39426920 | 96.21% | 93.41% | 34508122 (95.83%) |
| B2 | 49659178 | 48322660 | 96.33% | 93.63% | 42305696 (95.74%) |
| B3 | 47730646 | 46674952 | 96.95% | 94.07% | 40703747 (95.85%) |
| B4 | 37096236 | 36084022 | 96.25% | 93.48% | 31580844 (95.78%) |
| B5 | 37023098 | 36003418 | 96.19% | 93.39% | 31510718 (95.88%) |
| P1 | 45789972 | 44827634 | 97.08% | 94.21% | 38826139 (95.31%) |
| P2 | 38952696 | 37869280 | 96.24% | 93.45% | 32753828 (95.37%) |
| P3 | 36306716 | 35286550 | 96.16% | 93.32% | 30346600 (95.72%) |
| P4 | 40436352 | 39370226 | 96.36% | 93.67% | 34015734 (95.31%) |
| P5 | 43996068 | 43158596 | 97.01% | 94.39% | 36994705 (95.62%) |
| PB1 | 47169058 | 46077566 | 96.87% | 94.01% | 39758026 (95.40%) |
| PB2 | 46966758 | 46149994 | 97.27% | 94.57% | 40274990 (95.57%) |
| PB3 | 43486090 | 42470594 | 96.76% | 93.98% | 36486917 (95.83%) |
| PB4 | 47280186 | 46222866 | 96.87% | 94.04% | 39749450 (95.57%) |
| PB5 | 45485436 | 44527580 | 96.96% | 94.20% | 38971393 (95.86%) |

**Supplemental Table 8. Primers Used for Taxonomic Identification of Species**

| Number | Primer name | Primer sequence（5＇-3＇） |
| --- | --- | --- |
| 1 | 16S -27F | AGAGTTTGATCMTGGCTCAG |
|  | 16S -1492R | GGYTACCTTGTTA CGACTT |
| 2 | *gyrB*-F | AGCAGGGTACGGATGTGCGAGCCRTCNACRTCNGCRTCNGTC |
|  | *gyrB*-R | GAAGTCATCATGACCGTTCTGCAYGCNGGNGGNAARTTYGA |

**Supplemental Table 9. Primer information for qPCR.**

| Primer name | Primer sequence (5' to 3') |
| --- | --- |
| actin-F | AATGACGCAGATTATGTT |
| actin-R | GAATCCATCACAATACCA |
| Zm00001d002309-F | CAAGAATTACTTAACAGGTT |
| Zm00001d002309-R | AGAGATACAAGATTCACAA |
| Zm00001d002288-F | TGAATCTTACCGTCCAAT |
| Zm00001d002288-R | CAATCTAACAAGTAGTAGCATA |
| Zm00001d041204-F | CCAACAGCGTCTCCATAT |
| Zm00001d041204-R | AACCGACAACCATAATCAGA |
| Zm00001d029801-F | CATTGGCGATTGTTCATTGTGTAA |
| Zm00001d029801-R | ATGGAGGCGACGAGTTCA |
| Zm00001d022550-F | CTTGCTTGCTCAGGTAAA |
| Zm00001d022550-R | GGAGAATCTTGCGAACAA |
| Zm00001d010667-F2 | ATCGTGTTTGCCTGGTTGAC |
| Zm00001d010667-R2 | CATAAACCACAGCGATGCGA |
| Zm00001d031697-F2 | TGTATGCCATGTCCGGATGA |
| Zm00001d031697-R2 | CACACATCCTGCGTCTGAAG |
| Zm00001d002599-F2 | GCCAGTTTGACGAGGAGAAC |
| Zm00001d002599-R2 | CCTCTTTCCTGAACGACCCT |
| Zm00001d048453-F3 | CTTTTGAGAACGGCCTGTCC |
| Zm00001d048453-R3 | AACTCATCCTGGCCTCTCAC |
| Zm00001d014657-F | GCTTCACTGTCCATCTCTG |
| Zm00001d014657-R | GCCTCACCATCGTTCTTG |
| Zm00001d045190-F | GCAGATTACCGAGGCTAC |
| Zm00001d045190-R | TGTTGTGTTCAGATATGTTGTC |

## Supplementary Figures


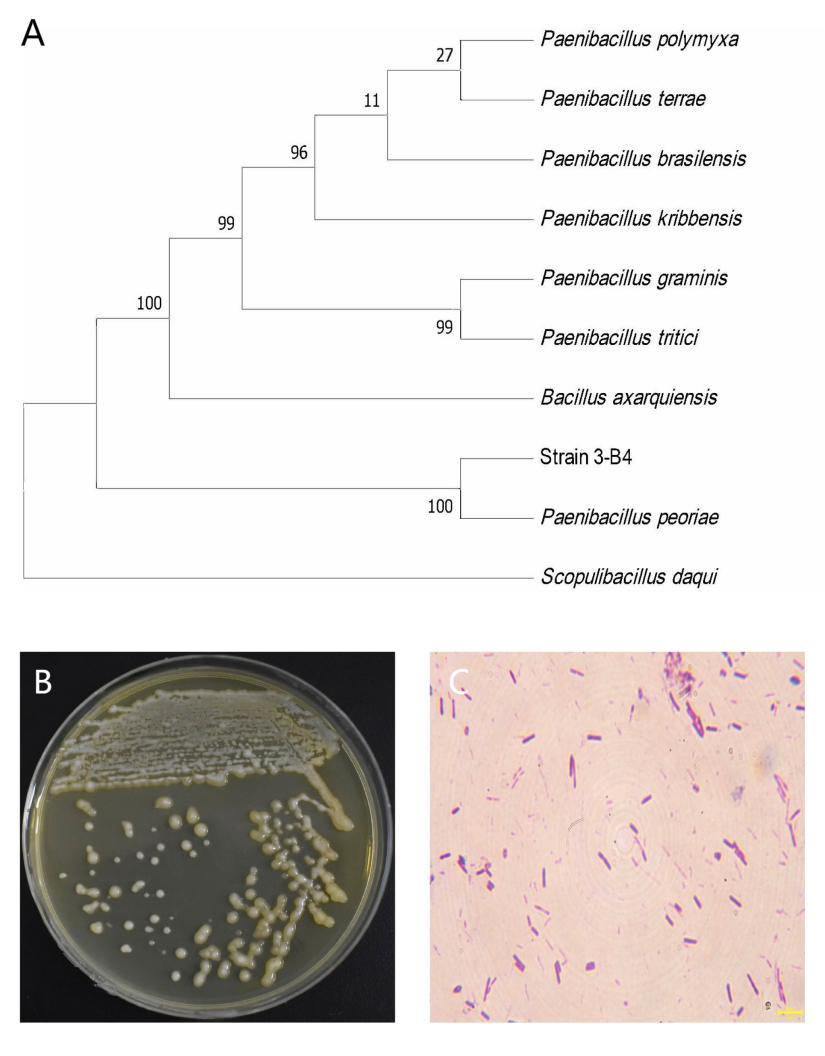


**Supplementary Figure 1.** **Morphological and molecular identification of antagonistic strains of *Paenibacillus peoriae* 3-B4.** (A) The phylogenetic tree of *P. peoriae* 3-B4. (B) The colony morphology of P. peoriae 3-B4. (C) The Gram stain observations of *P. peoriae* 3-B4.


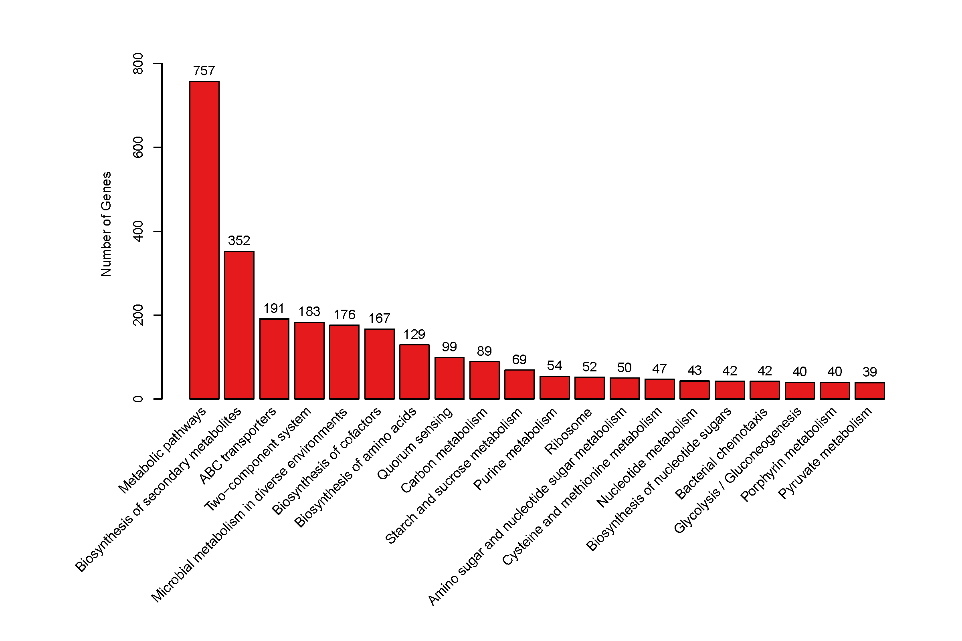


**Supplemental Figure 2. Top 20 KEGG annotated pathways.** Each bar represents a different category of biometabolic and cellular processes, and its length and value indicate the percentage of genes in that category.

**
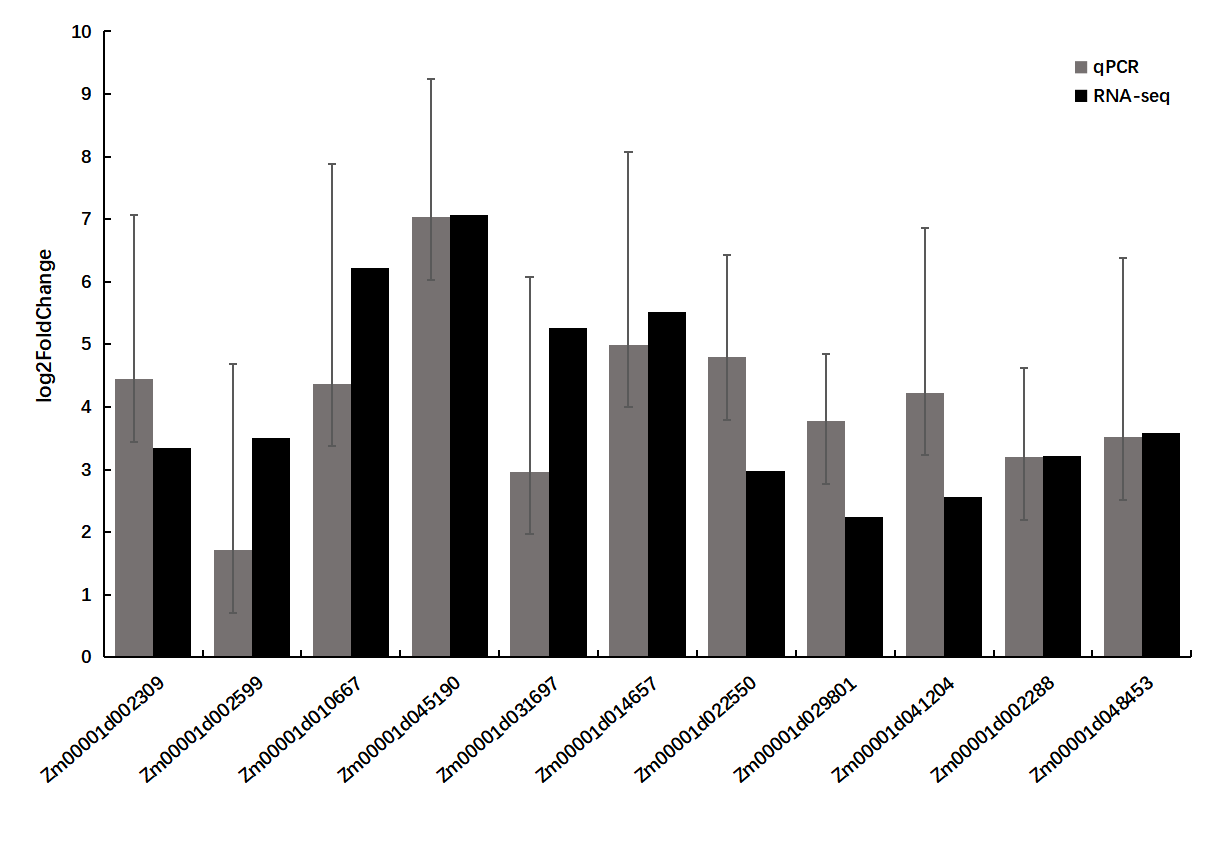
**

**Supplemental Figure 3. Quantitative PCR (qPCR) analysis.**
